# Molecular Modeling Studies of *N*-phenylpyrimidine-4-amine Derivatives for Inhibiting FMS-like Tyrosine Kinase-3

**DOI:** 10.3390/ijms222212511

**Published:** 2021-11-19

**Authors:** Suparna Ghosh, Seketoulie Keretsu, Seung Joo Cho

**Affiliations:** 1 Department of Biomedical Sciences, College of Medicine, Chosun University, Gwangju 501-759, Korea; s.ghosh@chosun.kr (S.G.); keretsu@chosun.kr (S.K.); 2 Department of Cellular and Molecular Medicine, College of Medicine, Chosun University, Gwangju 501-759, Korea

**Keywords:** FMS-like tyrosine kinase-3, acute myeloid leukemia, MD simulation, binding free energy, 3D-QSAR, CoMFA, CoMSIA

## Abstract

Overexpression and frequent mutations in FMS-like tyrosine kinase-3 (FLT3) are considered risk factors for severe acute myeloid leukemia (AML). Hyperactive FLT3 induces premature activation of multiple intracellular signaling pathways, resulting in cell proliferation and anti-apoptosis. We conducted the computational modeling studies of 40 pyrimidine-4,6-diamine-based compounds by integrating docking, molecular dynamics, and three-dimensional structure–activity relationship (3D-QSAR). Molecular docking showed that K644, C694, F691, E692, N701, D829, and F830 are critical residues for the binding of ligands at the hydrophobic active site. Molecular dynamics (MD), together with Molecular Mechanics Poison–Boltzmann/Generalized Born Surface Area, i.e., MM-PB(GB)SA, and linear interaction energy (LIE) estimation, provided critical information on the stability and binding affinity of the selected docked compounds. The MD study suggested that the mutation in the gatekeeper residue F691 exhibited a lower binding affinity to the ligand. Although, the mutation in D835 in the activation loop did not exhibit any significant change in the binding energy to the most active compound. We developed the ligand-based comparative molecular field analysis (CoMFA) and comparative molecular similarity index analysis (CoMSIA) models. CoMFA (*q*^2^ = 0.802, *r*^2^ = 0.983, and $Q_{F3}^{2}$ = 0.698) and CoMSIA (*q*^2^ = 0.725, *r*^2^ = 0.965 and $Q_{F3}^{2}$ = 0.668) established the structure–activity relationship (SAR) and showed a reasonable external predictive power. The contour maps from the CoMFA and CoMSIA models could explain valuable information about the favorable and unfavorable positions for chemical group substitution, which can increase or decrease the inhibitory activity of the compounds. In addition, we designed 30 novel compounds, and their predicted pIC_50_ values were assessed with the CoMSIA model, followed by the assessment of their physicochemical properties, bioavailability, and free energy calculation. The overall outcome could provide valuable information for designing and synthesizing more potent FLT3 inhibitors.

## 1. Introduction

**Figure 1:**
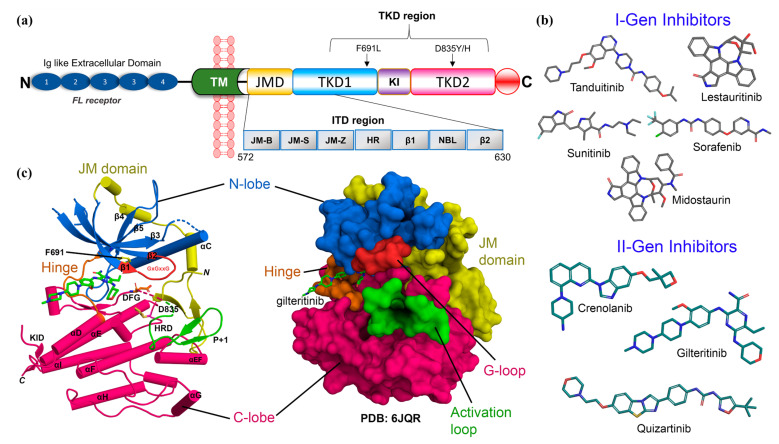
Domain map, inhibitors, and X-ray structure of FLT3. (**a**) Full-length FLT3 contains five immunoglobulins (Ig) like domains at the N-terminal, a transmembrane (TM) domain, and a juxtamembrane domain (JMD), followed by the tyrosine kinase domain (TKD) with a kinase insert (KI) and a C-terminal intracellular domain. F691L and D835Y/H are the two widely reported mutations in the TKD region, reported for 5% of the total AML cases. (**b**) FDA-approved first generation (I-Gen) and second generation (II-Gen) drugs as FLT3 inhibitors in grey and deep teal C atom color scheme. (**c**) X-ray structure of the gilteritinib-bound FLT3 (PDB: 6JQR). The various subregions of FLT3 are shown in cartoon and surface representation.

FLT3 belongs to the type III tyrosine kinase receptor together with KIT, FMS, and platelet-derived growth factor receptor (PDGFR), which are involved in the differentiation, proliferation, and survival of hematopoietic progenitor cells [1]. It is expressed by the stromal cells of bone marrow, placenta, and CD34^+^ cells, as well as the myeloid and B lymphoid cell lineage [2]. The FLT3 structure comprises five extracellular domains similar to immunoglobulin (Ig) at the N-terminal, followed by a single transmembrane (TM) domain, a cytoplasmic juxtamembrane domain (JMD), and a tyrosine kinase domain (TKD) separated by a kinase insert (KI). An intracellular domain was located at the C-terminal end, as shown in Figure 1a [3,4,5]. FL is the endogenous ligand of FLT3, which is also expressed in bone marrow stromal cells, exists in a soluble form, or is bound to the membrane. Predominantly, FLT3 is found in an unbound form as an unphosphorylated monomer coordinated by an inactive kinase domain. The JM domain interacts with the KD to block the ATP binding to the active site. After binding to the ligand FL in the extracellular Ig domains, FLT3 undergoes dimerization and exposes its dimerized domain [6,7,8]. This event triggers the activation of tyrosine kinase, followed by the phosphorylation of the FLT3 intracellular domain at its various sites. The phosphorylation of FLT3 propagates multiple intracellular signaling pathways, which are essential for phospholipid metabolism, transcription, differentiation, proliferation, cell survival, and apoptosis [9].

FLT3 is overexpressed in patients with AML, which is considered an aggressive hematologic malignancy. Active mutations in FLT3 have been reported in ~30% of total AML cases. These mutations can be subdivided into internal tandem duplicates (FLT3-ITD) and point mutations in the tyrosine kinase domain (FLT3-TKD), which are approximately 25% and 5% of the total AML, respectively [10,11]. Therefore, inhibition of FLT3 is an ideal therapeutic choice. Many inhibitors have been subjected to preclinical and clinical trials and have shown promising results. As illustrated in Figure 1b, tyrosine kinase inhibitors such as Tandutinib, Sunitinib, Midostaurin, Lestaurtinib, and Sorafenib were used as first-generation FLT3 inhibitors [12]. Due to the lack of sensitivity and selectivity of first-generation inhibitors, second-generation inhibitors, such as Gilteritinib, Quizartinib, and Crenolanib, have been approved [13,14]. Although, their poor bioavailability and off-target effects often increase drug toxicity in patients, which remains a concern. Patients undergoing AML treatment have often developed resistance to lead compounds through on-target secondary mutations in the kinase domain [13]. Two of the most common mutations have been found in the gatekeeper residue F691L and the activation loop D835Y, as shown in Figure 1c. Mutations in residues I836, D839, and Y842 are also found in the FLT3 kinase domain in patients with AML [14].

Inevitably, the rational development of new FLT3 inhibitors based on existing lead compounds is an ideal choice for achieving therapeutic efficacy in AML. Computer-aided drug design (CADD) has emerged as a promising tool for discovering new FLT3 inhibitors [15]. In a prior study, Bensinger et al. [16] used virtual screening and docking to identify lead compounds that were covalently bound to the DGF-in conformer of FLT3 receptor. These lead compounds were chemically modified to increase cytotoxicity and inhibitory efficacy against wild-type and mutant (D835Y) FLT3, suggesting that they could be a suitable starting point for discovering irreversible inhibitors [16]. In another study by Smith et al. [17], residue D835 plays a critical role in maintaining the DFG-out configuration by acting as an amino terminal capping residue for the αC-helix and serving as an essential space for type II inhibitor binding. However, the mutations in the D835 remain sensitive to type I inhibitors.

In this paper, we performed computational modeling studies such as docking, molecular dynamics (MD), free energy calculation, and three-dimensional structure-activity relationship (3D-QSAR) to a series of 40 pyrimidine-4,6-diamine derivatives, which are reported as type II-like inhibitors of FLT3 by Bharate et al. [18]. In their docking study, the most active compound 13a bound to the hinge loop by forming two H-bond interactions with C694. The compounds exhibited a diverse inhibitory activity range (IC_50_ 13.9 nM-15111 nM) against FLT3. The molecular docking and molecular dynamics revealed the critical interactions with the inhibitors inside the binding pocket. We calculated the MM-PB(GB)SA and LIE binding energy terms to evaluate the protein–ligand binding affinity. We manually induced the F691L and D835Y mutations in FLT3 and performed MD simulations to understand the mutagenic effect in terms of binding affinity to the inhibitor compound. Finally, we conducted CoMFA and CoMSIA studies of the 40 compounds to produce the 3D-QSAR contour map and established the structure–activity relationship. The CoMFA and CoMSIA contour maps described how modification of chemical groups could enhance the inhibitory activity of the compounds.

## 2. Results and Discussion

### 2.1. Molecular Docking Analysis

**Figure 2:**
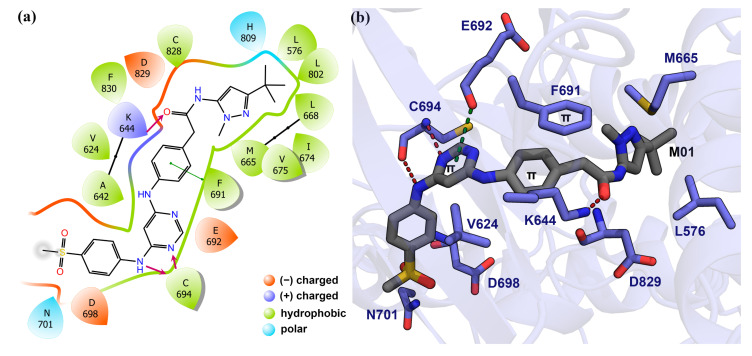
Molecular docking analysis of compound M01 and FLT3. (**a**) Compound M01 surrounded by the active site residues. The residues are illustrated based on their chemical properties in 2D representation. H-bonds are shown by magenta arrows. (**b**) Docking interaction of M01 with FLT3 residues. The H-bond interactions are shown by red dashed lines. The π–anion interaction is shown by green dashed lines.

As we conducted the MD simulation and 3D-QSAR study based on the docking pose, the verification of docking reliability is an important step. There were no compounds from the dataset available with the co-crystallized FLT3 form in the PBD database. Thus, we selected the *N*-phynylpyrimidine-4-amine substructure of the docked compounds and compared it with the existing FLT3 conjugated crystal ligand FF-10101 (PDB ID: 5X02) [19], and Quizartinib (PDB ID: 4XUF) [20], and AWO with C-kit (PDB ID: 6ITT) [21] by overlapping them in the ligRMSD server provided in Supplementary Table S1. Since FLT3 and C-kit both are members of the tyrosine kinase family, we opted for the AWO bound C-kit to compare the ligand interactions. The FF-10101 formed the critical H-bond interaction with residue C694 by the amine(-NH_2_) group of *N*-phenylpyrazine-2-amine. For the ligand Quizartinib, π–π interactions were found between its aniline ring and residue F691 and residue F830. We found the critical H-bond interaction between C694 and -NH_2_ of the *N*-(Pyrimidin-4-yl)thiazol-2-amine moiety, which anchored the ligand to the hinge loop. Similar interactions were observed with the docked compounds M01, M03, and M17. Compounds M20, M24, and M34 were anchored to the hinge loop by forming H-bond interactions between the residue C694 and the *N*-phenylpyrimidine-4-amine-NH_2_ group and the π–π stacking with phenylalanine (F691). The final docked pose was selected from the lowest binding energy cluster, which also shared the lowest RMSD with the crystal substructure according to the ECIDALs norms [22,23]. The final RMSD values of the docked compounds from the crystal ligands were arranged and are shown in Supplementary Table S1. The RMSD values suggest the overall docking reliability of the selected compounds. The BEs from the docking study were estimated to be −11.31 kcal/mol, −11.68 kcal/mol, −9.88 kcal/mol, −10.54 kcal/mol, −9.68 kcal/mol, and −10.09 kcal/mol for compounds M01, M03, M17, M20, M24, and M34, respectively. The 2D protein–ligand interaction is illustrated in Figure 2a. The surrounding residues within 3.5 Å are shown using a color scheme, which is based on the chemical properties of the amino acids. Figure 2b depicts the H-bond, π–π, and π–anion interactions between M01 and residues inside the ATP pocket of FLT3. With residue C694, two H-bond interactions were found, while a third H-bond interaction was observed with catalytic lysine K644. The gatekeeper residue E692 formed the π–anion interaction with the pyrimidine ring. Another gatekeeper residue F691 accomplished the π–π stacking with the phenyl ring of M01. Other residues, such as L616, M665, V675, L856, L576, and I801, participated in hydrophobic interaction inside the hydrophobic cleft between the N lobe and the C lobe. The docked complexes of compounds M03, M17, M20, M24, and M34, which also comply with the ECIDALs norms during the docking process, are shown in Supplementary Figure S2 by 2D interaction diagrams. The best performing protein–ligand single-docked coordinate of each complex was taken for further MD simulation study.

### 2.2. MD Analysis

**Figure 3:**
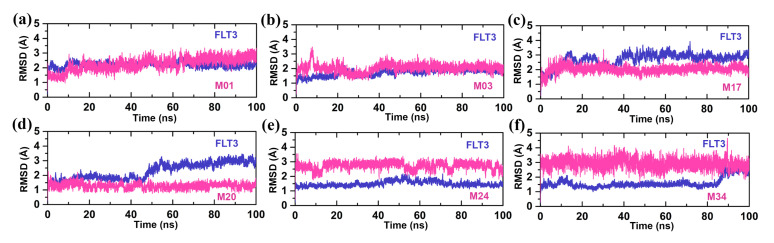
(**a**) FLT3-M01, (**b**) FLT3-M03, (**c**) FLT3-M17, (**d**) FLT3-M20, (**e**) FLT3-M24, and (**f**) FLT3-M34 are the RMSD graphs of protein–ligand complexes. The protein and ligand RMSDs are shown in slate and pink colors, respectively.

**Figure 4:**
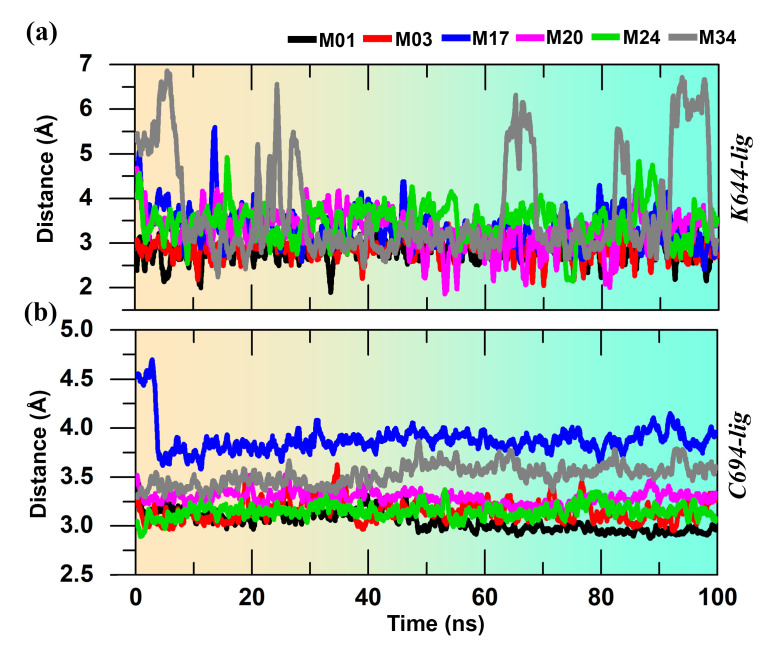
H-bond interaction distance analysis from (**a**) residue K644 and (**b**) C694 to the ligands during the production simulation.

**Figure 5:**
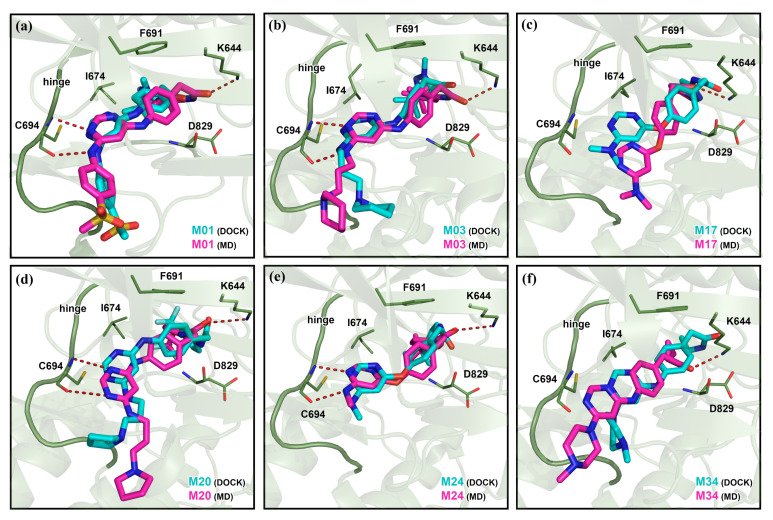
Average MD pose of the ligand at the active site of FLT3. The last 1 ns average MD poses of (**a**) M01, (**b**) M03, (**c**) M17, (**d**) M20, (**e**) M24, and (**f**) M34 inside the binding pocket are shown in pink color. The corresponding docked pose is shown in cyan color. The H-bond interactions are shown by red dashed lines.

The MD simulations were conducted to evaluate the overall stability of the protein–ligand complexes. During the 100 ns MD run, the protein–ligand complexes achieved convergence within the first 10 ns. The RMSDs of proteins were found in the range of 1.0–4.0 Å, and the RMSDs of ligands were found in the range of 0.5–4 Å, as shown in Figure 3. We observed a low RMSD for compounds M01, M03, and M20, while compounds M17, M24, and M34 had a higher RMSD. In particular, the RMSD of the compounds M24 and M34 reached 4 Å during the simulation. Since the two H-bond interactions with residues K644 and C694 played a critical role in the binding of the ligand inside the ATP pocket, we compared these two interacting H-bond distances for 100 ns in Figure 4. For compound M34, the H-bond distance with K644 was found to be unstable and a major fluctuation was observed, in contrast to the other compounds. Overall, this specific H-bond distance was found to be within the range of 2–3.8 Å in Figure 4a. When comparing the H-bond distance between the ligands and N atom of residue C694, compounds M17 and M34 showed a slightly higher H-bond distance to ~3.7 Å in Figure 4b. However, this H-bond interaction was intact for the remaining compounds. Following that, we compared the average MD structure of protein–ligand complexes from the last 1 ns to their final docked position in Figure 5. Figure 5a,b,e shows that *N*-phenylpyrimidine-4-amine substructures of M01, M03, and M24 closely retained their docking pose and molecular interactions until the end of the MD run. For M17, we observed that the dimethylamine with the pyrimidine ring was displaced from its initial docked position, which might increase the critical distance for H-bond formation as shown in Figure 5c. Compound M20 formed the H-bond interactions to the C694 with its N atoms of the pyridine ring, while another H-bond interaction remained intact with K644 (Figure 5d). The piperazine ring in M34, on the other hand, shifted forward from its initial docking position inside the ATP pocket (Figure 5f), which could be a reason for the extension of the distance from residue C694. We also observed the rotational and translational displacement of the piperidine ring and the pyrrolidine ring of compounds M03 and M20 by comparing their respective MD trajectories and docking positions.

### 2.3. MM-PB(GB)SA and LIE Estimation

**Figure 6:**
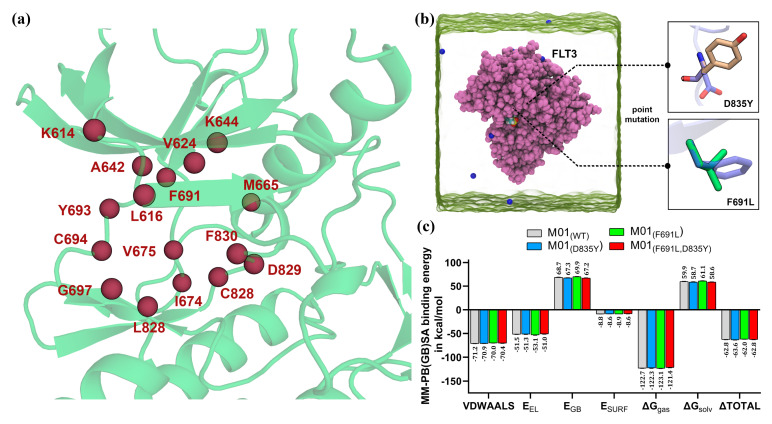
MM-PB(GB)SA binding free energy comparison between M01 and FLT3 variants. (**a**) Common residues that contributed to the ΔTOTAL BE to the ligands are shown in the sphere representation. (**b**) Point mutations were manually performed at D835 and F691 in Pymol to compare the MM-PB(GB)SA binding energy. (**c**) Comparison of the MM-PB(GB)SA binding energy terms between WT, D835Y, F691Y, and both (F691L, D835Y) mutant FLT3 and M01.

We implemented the MM-PB(GB)SA and LIE estimation to investigate the binding affinity of FLT3 to selected ligands. To calculate the MM-PB(GB)SA and LIE binding energy, we used the final 2 ns or 200 frames of each protein–ligand MD trajectory. The final ΔTOTAL BEs were found to be −62.80, −60.27, −47.32, −60.68, −59.56, and −49.31 kcal/mol for compounds M01, M03, M17, M20, M24, and M34, respectively. The final ΔG_bind_ from the LIE estimation was found to be −18.39, −13.43, −12.56, −21.37, −9.75, and −8.14 kcal/mol for compounds M01, M03, M17, M20, M24, and M34, respectively. The detailed MM-PB(GB)SA energy and LIE terms are shown in Table 1. We estimated the per-residue BE decomposition by selecting residues within 4.0 Å from the ligand atoms. The common residues with positive or negative BE decomposition are compared in Table 2. We found that residues L616, V624, A642, K644, M665, I674, V675, F691, Y693, C694, G697, L818, C828, D829, and F830 were the key interacting common residues, contributing the critical BE to the ligands shown in Figure 6a. We acquired the substantially equilibrated 80th ns wild-type (WT) FLT3-M01 complex and manually mutated D835Y, F691L, and both (D835Y and F691L) at the same time. The newly prepared three different FLT3-M01 complexes were subjected to standard 20 ns MD simulations, followed by MM-PB(GB)SA and LIE calculations from the last 2 ns trajectory. The ΔTOTAL binding energy of MM-PB(GB)SA was found to be −63.55, −62.03, and −62.79 kcal/mol for the D835Y, F691L, and both mutants of FLT3 variants to compound M01, respectively. However, we were unable to observe any significant changes in ΔTOTAL BE terms with M01. During the calculation process, we restricted the analysis of per-residue MM-PB(GB)SA decomposition within the 4.0 Å distance from the ligand atoms. The per-residue BE decomposition between the F830 of FLT3 mutants and compound M01 was not generated, suggesting that the distance between M01 and F830 increased to more than 4.0 Å, whereas the appearance of BE decompositions from residue K614 signify its proximity to compound M01.

However, rather lower free energy values were observed in the VDWAALS and E_EL_ energy terms with the FLT3_(F691L)_-M01 complex, as shown in Figure 6b,c. Minor energy differences could occur due to the substitution of the bulky hydrophobic residue F691 with a non-bulky leucine residue, as F691 is the key gatekeeper residue and had a strong π–π interaction with the phenyl ring of M01. The LIE values were found to be −18.39, −17.75, and −17.95 kcal/mol for F691L, and both mutant variants of FLT3 to compound M01, respectively. Residue D835Y mutation in the activation loop was 14.1 Å away from the active site. This mutation in the DFG-out FLT3 did not influence the final BE to the type II-like M01 in the estimation of MM-PB(GB)SA or LIE. Therefore, we assumed that the F691L mutation could partially affect selectivity and ligand interaction by changing the hydrophobic property of the nucleotide-binding pocket. This could also decrease the relative competitiveness of M01 against ATP and induce drug resistance [24,25]. In contrast, the D835Y mutation in the activation loop is strongly associated with the resistance mechanism of the active conformation (DFG-in) tyrosine kinase inhibitors (TKI). A pathway of the allosteric network has been proposed between the activation loop and the DFG motif [26], which plays an important role in stabilizing the adenosine triphosphate (ATP) molecule by chelating the Mg^2+^ ions at the catalytic site. The D835Y mutation could trigger an alteration of the allosteric mechanism in FLT3 by forming a dead mutant of kinases or disfavoring inhibitor binding by increasing entropy, ultimately influencing the entry of the ligand into the ATP-binding pocket [27].

### 2.4. Dataset Building, 3D-QSAR Model Development, and Model Validation

**Figure 7:**
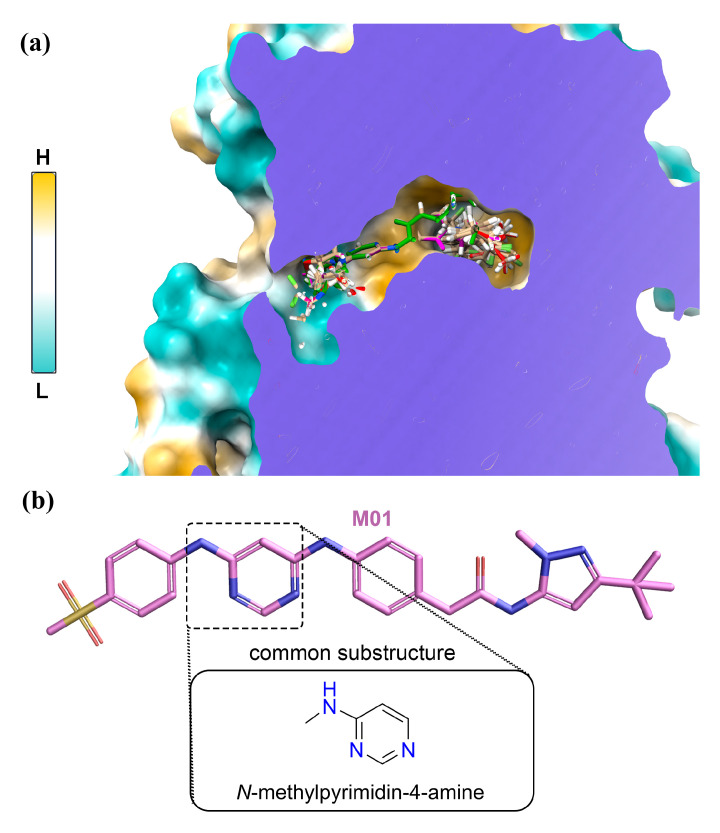
Common substructure-based molecular alignment. (**a**) Alignment of the compounds is shown inside the hydrophobic binding pocket of FLT3 by Z-plane clipping. Cyan to gold-yellow color bar indicate low (L) to high (H) hydrophobicity. (**b**) Common substructure of the compounds in the dataset.

Coherence selection of the training set and test set compounds is a vital step toward the development of a 3D-QSAR statistical model. The chemical structures and their respective pIC_50_ values, which were well spanned over three log units, are illustrated in Supplementary Table S2. Initially, we classified the compounds into high-, medium-, and low-activity groups. Next, we chose compounds at random from each group to form the test set while maintaining structural diversity. We developed two sets of 3D-QSAR models, and their statistical validation is summarized in Table 3. The final 1 ns MD average structure of compound M01 was regarded as a 3D bioactive conformer and selected as a template molecule, as described here [28]. The rest of the compounds were modeled based on the template molecule and aligned with the common skeleton of *N*-methylpyrimidine-4-amine. The maximum common substructure (MCS) functionality was used to find the common core. The alignment of the dataset compounds inside the hydrophobic cleft and their unique chemical core are shown in Figure 7a,b. The first model was built by taking 40 compounds altogether. We strictly adhered to the statistical parameters listed in the threshold values column, as well as those described in the Methodology Section when validating each model.

**Figure 8:**
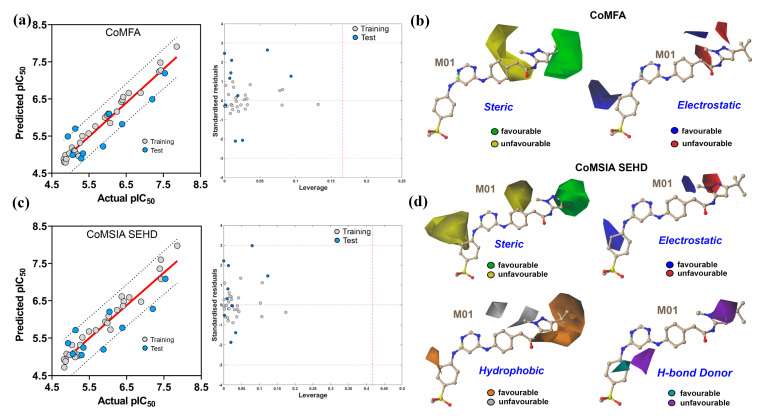
PLS scatter plot, Williams plot, and CoMFA-CoMSIA contour maps analysis. (**a**) Scatter plot of actual vs. predicted pIC_50_ values from the CoMFA and the corresponding Williams plot for AD analysis. (**b**) The green and yellow contours from CoMFA indicate the favorable and unfavorable substitution for the steric groups. The blue and red contours indicate the favorable and unfavorable positions for the electropositive groups. (**c**) Scatter plot of actual vs. predicted pIC_50_ values from the CoMSIA and the corresponding William’s plot for AD analysis. (**d**) From CoMSIA, green and yellow contours signify the favorable and unfavorable positions for the steric group, whereas the blue and red contours show the favorable and unfavorable substitutions for electropositive groups. The orange and gray contours show the favorable and unfavorable positions for the hydrophobic groups. The cyan and purple colors signify the favorable and unfavorable positions for the H-bond donor groups.

In the first 3D-QSAR model, we obtained the best *q*^2^ and *r*^2^ values of 0.735 and 0.956, respectively, at an ONC of 6 in the CoMFA scheme. The SEHA produced the best statistical CoMSIA model with *q*^2^ and *r*^2^ values of 0.725 and 0.912, respectively at an ONC of 5 among the different combinations of descriptor fields (Supplementary Table S3). The *q*^2^ and *r*^2^ values of both CoMFA and CoMSIA appeared to be greater than the accepted threshold value (*q*^2^ > 0.5 and *r*^2^ > 0.6), which indicated a good agreement for the internal validation of both models. The χ^2^ and RMSE values were found to be 0.052 and 0.219, respectively. In CoMSIA, the χ^2^ and RMSE values were found to be 0.078 and 0.265, respectively. The χ^2^ and RMSE values are within the acceptable threshold value, which signifies the fitness and accuracy of the models. However, any QSAR model would be uncertain without being validated externally. Therefore, the second model was developed using the training set of 30 compounds. The remaining 10 compounds were used as a test set to analyze the external predictivity. In the second 3D-QSAR model, the training set compounds generated the best *q*^2^ and *r*^2^ values of 0.802 and 0.960, respectively, at an ONC of 6 in the CoMFA scheme. The χ^2^ (χ^2^ = 0.012) and RMSE (RMSE = 0.119) values were determined within the acceptable range of <0.3 and <0.5 [29], indicating for a good internal validation and model’s fitness. For external validation, we calculated multiple statistical parameters described in these studies [30,31,32]. The *k* and *k*′ were found to be 1.033 and 0.962, and the *|r*_0_^2^−*r*′_0_^2^*|*, (*r*^2^−*r*_0_^2^)/*r*^2^, *r_m_*^2^, and Δ*r_m_*^2^ were predicted to be 0.109, 0.041, 0.724, and 0.059, respectively. In addition, we acquired the $Q_{F3}^{2}$ and $Q_{ccc}^{2}$ metrics determination to evaluate the predictive ability of the CoMFA model. The $Q_{F3}^{2}$ value was found to be 0.698, which greater than the acceptable range (>0.6), and a high $Q_{ccc}^{2}$ value of 0.821 signifies the true predictive power of the model. To generate the best possible CoMSIA model, we applied five molecular descriptor fields—steric (S), electrostatic (E), hydrophobic (H), H-bond acceptor (H), and H-bond donor (D)—in different combinations (Supplementary Table S4). The actual and predicted activities with their residuals are tabulated in Supplementary Table S5. The combination of SHD produced the best statistical *q*^2^ values (*q*^2^ = 0.730) at an ONC of 5. However, the best *q*^2^ value does not necessarily indicate a good predictivity. Instead, the best CoMSIA model was selected based on the highest $Q_{F3}^{2}$ metrics value. We found that the SEHD combination produced *q*^2^ and *r*^2^ values of 0.725 and 0.965, respectively, at an ONC of 5. The model had the $Q_{F3}^{2}$ value of 0.665, the highest among all. Despite this, the model successfully passed all other statistical parameters. The *k* and *k*′ were found to be 1.033 and 0.961, and the *|r*_0_^2^−*r*′_0_^2^*|,* (*r*^2^−*r*_0_^2^)/*r*^2^, *r_m_*^2^, and Δ*r_m_*^2^ were predicted to be 0.109, 0.041, 0.724, and 0.059, respectively. Thereafter, the SEHD combination was selected as the final CoMSIA model. Overall, the second QSAR model, including CoMFA and CoMSIA (SEHD), produced the highest statistically significant values; therefore, we selected these models for contour map generation and SAR analysis. The PLS regression graph of CoMFA and CoMSIA is shown in Figure 8a,c, which described that our 3D-QSAR model could adequately predict the pIC_50_ values of the test set compounds. The contribution of the steric and electrostatic field was found to be 54.4% and 45.6% in the CoMFA model, respectively, while the contributions of the steric, electrostatic, hydrophobic, and H-bond donor were found to be 20.5%, 29.9%, 32.8%, and 16.8%, respectively, in the CoMSIA model. Both models established good agreement between experimental activity and predicted activity by showing acceptable criteria (*q*^2^ > 0.6, *r*^2^ > 0.8) while having a satisfactory predictive performance ($Q_{F3}^{2}$ > 0.5).

The progressive scrambling method was applied to evaluate the additional stability of the CoMFA and CoMSIA models, as shown in Table 4. We ran 100 independent scrambles with minimum and maximum bin sizes of 2 and 10. At component number 6, scrambling Q^2^ and cSDEP were measured to be 0.502 and 0.713, respectively. The d*q*^2^/d*r*^2^*_yy_*_′_ did not exceed the value 1.2 (d*q*^2^/d*r*^2^*_yy_*_′_ = 1.198) in the CoMFA. In the CoMSIA, the scrambling Q^2^, cSDEP, and d*q*^2^/d*r*^2^*_yy_*_′_ were found to be 0.518, 0.709, and 0.982, respectively. Progressive scrambling helps to identify the optimal number of components of the model. In addition, it detects the model’s sensitivity over a small perturbation when applied to the data.

The QSAR models are generated by a limited number of compounds and can predict inhibitory activity for an unknown chemical that has a very similar chemical constitution. To assess the reliability of the CoMFA and CoMSIA models and specify the outliers, we used the applicability domain analysis by distance-based Williams plot as described in previous work [33]. The CoMFA and CoMSIA PLS plots, and the corresponding Williams plots, portrayed the standardized residuals of the training set and test set compounds against their leverage values. Compounds with a high leverage (*h_i_*) value greater than the warning leverage (h*) can be detected as outliers and have a substantial impact on the fitness of the model. We used the *Applicability Domain toolbox*, a MATLAB package developed by Sahigara et al. [34] to perform the AD analysis. In our study, all the compounds fell within the ±3 standardized residuals and the estimated warning leverages in CoMFA (h* = 0.16) and CoMSIA (h* = 0.42), confirming the overall predictive reliability of the models.

### 2.5. CoMFA and CoMSIA Contour Map Analysis

We generated 3D contour maps around the most active compound M01 in the SYBYL-X2.1 suit to elucidate the structure–activity relationships. The information collected from the contour map could be useful to improve the inhibitory potency of small molecules by altering the chemical groups. Figure 8b displayed the contour maps of the CoMFA analysis. The green and yellow contours around the pyrazole ring represent the favorable and unfavorable substitutions for the bulky and steric chemical groups. Specifically, the location of the green contour arrived near the residues M665 and V675, with which M01 formed the hydrophobic interaction in the docking study, and these residues contributed a BE of −1.48 and −1.81 kcal/mol, respectively. Compounds M01–M06, M11, and M38–M40 had steric groups in their R_2_ position at the green contour, which could explain why its inhibitory activity is higher than that of compounds M31–M35.

In contrast, compounds M07, M08, and M10 had the steric phenyl ring facing the yellow contour at the R_2_ position, which could decrease their inhibitory potency due to the probability of the steric hindrance effect. In Figure 8b, the blue and red contours show the favorable positions for the electropositive and electronegative groups. Compounds M01–M05, M38–M39, and M40 had the N atom in their pyrazole ring in the R_2_ position and exhibited a higher inhibitory activity. Besides, a blue contour near the red contours at the R_2_ position was found to show an unfavorable position for an electronegative group. Compounds M12–M16, M36, and M37 bearing the oxygen atom on their isoxazole ring and compounds M23, M27, M29, and M30 bearing the electronegative fluorine atom in the position R_2_ exhibited a lower inhibitory activity than M01–M05. For the same reason, compound M40 had less inhibitory potency than compound M01. Another blue contour was present near the phenyl ring at the R_1_ position, showing the favorable position of an electropositive chemical group. For compounds M15, M22, M24, and M35, an electropositive group was absent and showed lower inhibitory activity than compound M16.

Figure 8d illustrates the contour maps from the CoMSIA model analysis. We found that the steric and electrostatic descriptors yielded contour maps similar to those produced by CoMFA model. As a result, they are not discussed further. It also provided additional information on the hydrophobic and H-bond donor descriptors fields, making it preferred for SAR study. The favorable and unfavorable substitutions for hydrophobic chemical groups are shown by an orange-gray color scheme. A large orange contour at position R_2_ around the pyrazole ring indicated that a hydrophobic chemical group would be beneficial in increasing inhibitory activity. In compounds M18–M21, M25, and M28, the methyl groups were replaced by fluorine atoms, and the presence of the methylsulfonyl group in M22 may affect the inhibitory activity of these compounds.

**Figure 9:**
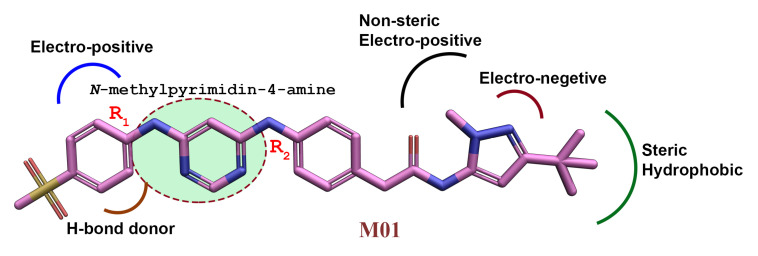
The three-dimensional structure–activity relationship by taking compound M01 as a reference.

Similarly, the favorable and unfavorable positions for the H-bond donor groups are shown by cyan and purple contours. For this reason, compound M36 had an N atom in the R_2_ position toward the purple contour and exhibited less inhibitory activity. From the analysis of the CoMFA and CoMSIA contour maps, we summarized an ideal SAR scheme in Figure 9. A positive charge group and an H-bond donor group on the methyl phenyl sulfone ring at position R_1_ could enhance the potency of M01. At that position, residue C694 formed two H-bond interactions with the M01 and contributed−2.82 kcal/mol MM-PB(GB)SA ΔTOTAL binding free energy. As a result, a hydrophobic group next to this residue would most likely hinder the formation of an H-bond, lowering the binding free energy decomposition.

Besides, the substitution of a smaller electron-donating group and the addition of a bulky hydrophobic group on the methylpyrazole ring in R_2_ could improve the bioactivity of the compounds. However, the docking and MD study suggested that the steric substitution at the R_2_ position was not infinite. A larger hydrophobic substitution could cause steric clashes with the hydrophobic residues in the αC-helix. Thereby, a smaller chemical group with steric and hydrophobic properties would be the preferred alteration strategy for an efficient interaction.

### 2.6. Designing of New Compounds

In the context of SAR analysis, we designed 30 new compounds using M01 as a template and evaluated their predictive activity in the CoMSIA-SHED model (Supplementary Table S6). Of these, 16 designed compounds were predicted to have higher inhibitory activity (pIC_50_) than the most active compound M01. We re-docked these compounds against FLT3 and determined their physicochemical property and SA score (Supplementary Table S7). An SA score predicts the difficulty level in synthesizing the unknown chemical compounds. An SA score of 1 indicates the easy synthesis route, while an SA score of 10 signifies the difficult synthesis route. The SA score of the newly proposed compounds ranged from 3 to 5, suggesting that the compounds would be simple to moderately challenging to synthesize. To ensure that the new compounds would be viable lead compounds, we further assessed the ADMET properties (Supplementary Table S8). The absorption value of more than 70% revealed that all compounds could be well absorbed in the human intestine. The LogBB > −2 and LogPS < −3 signify a better blood–brain barrier and CNS permeability. All the designed compounds were shown to be permeable through the BBB and CNS, while some of the compounds, such as D01, D02, D09–D12, and D17 may have a poor, yet acceptable CNS permeability. The cytochrome P450 enzymes are involved in the metabolism and biotransformation of the drug-like compounds. Except for D22, the other designed compounds are predicted to be substrates and inhibitors of CYP3A4. Excretion can be described as a constant ratio between drug concentration and renal clearance from the human body. The designed compounds yielded the acceptable values of total clearance, which were given in log mL/min/kg unit. Additionally, none of the designed compounds was predicted to be toxic in the AMES test.

**Figure 10:**
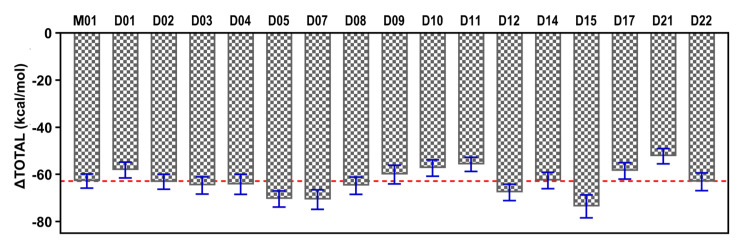
Comparison of the final ΔTOTAL binding energy terms of the newly designed compounds. Compounds D02–D08, D12, D15, and D22 were estimated to have higher binding free energy compare to M01 (red dashed line) and the standard deviation sign is shown in blue.

The designed compounds with FLT3-bound complexes converged well within the 50 ns of the MD simulation (Supplementary Figure S3); therefore, they were not extended further. We calculated the MM-PB(GB)SA binding free energy of the last 2 ns of each trajectory (Table 5 and Figure 10) and per-residue binding free energy decomposition (Supplementary Table S9). The designed compounds such as D02–D08, D12, D15, and D22 showed a higher binding free energy compared to the most active compound. The last 1 ns average structure of each complex is illustrated in Supplementary Figure S4. The results strongly suggest that SAR-assisted newly designed compounds could have a better binding affinity for FLT3.

## 3. Materials and Methods

### 3.1. Protein Structure Preparation and Molecular Docking

The FLT3 protein structure with a resolution of 2.20 Å was retrieved from the Protein Databank (PDB: 6JQR) [8]. The water molecules and ligands from the crystallographic solution were removed from the protein crystal. The missing residues were remodeled using Modeler-10.1 (University of San Francisco, San Francisco, CA, USA) in UCSF Chimera-1.14 (RBVI, UCSF, San Francisco, CA, USA), and the final model was endorsed in a DFG-out configuration. The model with the lowest DOPE score was selected and the entire protein structure was verified using the PROCHECK (DOE-MBI service, UCLA, Los Angeles, CA, USA) server (Supplementary Figure S1).

Compound M01 was the most active compound in the dataset and was thus chosen for the docking study, as described in the previous study [35,36,37]. Compounds M03, M17, M20, M24, and M34, with different subgroups at their positions R_1_ and R_2_ exhibiting a diverse range of activity, were also included for the docking study. Briefly, the protein was prepared by assigning polar hydrogens and Kollman charges in AutoDockTools (AutoDock 4.2, Scripps Research, La Jolla, CA, USA). The ligand was sketched and minimized, and hydrogens were added in SYBYL-2.1, followed by the addition of Gasteiger charges. The number of rotatable bonds was fixed at six to the ligands to avoid conformational explosion. We assigned the active site according to the X-ray structure of Quizertinib-bound FLT3 (PDB:4XUF). The grid box size was set to 50 × 60 × 50 in the X, Y, and Z directions, respectively, with a grid spacing of 0.375 and a grid center of X = −34, Y = −10, and Z = −25 to compute the grid parameters using AutoGrid. For the conformational search, the Lamarckian Genetic Algorithm (LGA) was employed. Finally, AutoDock-4.2 was used to perform 100 docking runs. This protocol was repeated for the remaining compounds. AutoDockTools, Pymol (Schrodinger, Inc., New York, NY, USA), and Maestro (Schrodinger, Inc., New York, NY, USA) were used to analyze the docking results. All protein–ligand docked complexes were taken for the MD simulation study.

### 3.2. Molecular Dynamics

The MD simulation was carried out with GROMACS 2019.5 [38] using the Amber14SB [39] force field. The topology and parameters of the small molecules were generated using ACPYPE [40]. The system was prepared by placing the protein–ligand complex in a cubic periodic box. The complex was then wrapped with the TIP3P water model in such a way so that the minimum thickness of the water wall was maintained at 10 Å from the protein atoms. An adequate amount of NA^+^ and Cl^−^ ions were added to neutralize the system.

The steepest descent algorithm was applied for 10,000 steps to minimize the system, followed by 200 ps of constant substance, volume, and temperature (NVT) simulation using the modified Berendsen thermostat (V-rescale) to attain a temperature of 300 K. Next, a 400 ps constant substance, pressure, and temperature (NPT) simulation was executed using the modified Berendsen barostat (V-rescale) to achieve 1 bar of pressure. The backbone and heavy atoms of the ligands were kept restrained during the minimization, NVT, and NPT simulation steps. Finally, the system was subjected to the 100 ns MD simulation run by un-restraining the backbone and heavy atoms. The Particle Mesh Ewald (PME) scheme was employed to maintain the electrostatic interactions, and the SHAKE algorithm was employed to deal with the bond length constraints. The cut-off distance was set at 12 Å to calculate the coulombic and van der Waals (vdW) interactions, respectively. The protocol was followed for the rest of the docked complexes as described in the previous study [41]. RMSD and H-bond distances were calculated by the in-build ‘*gmx rms’* and ‘*gmx distance*’ functions in Gromacs.

### 3.3. MM-PB(GB)SA and LIE

The MM-PB(GB)SA is a useful technique for computing the end-state binding free energy between the protein and the ligand. In our study, the binding free energy of MM-PB(GB)SA was calculated using the gmx_MMPBSA [42] package based on MMPBSA.py [43]. The protein–ligand binding free energy of MM-PB(GB)SA can be expressed by Equation (1),

(1) $$\Delta G_{bind}=\Delta G_{complex}-\Delta G_{protein}-\Delta G_{ligand}\;$$

(2) $$\Delta G_{bind}=\Delta E_{MM}+\Delta G_{sol}-T\Delta S$$

(3) $$\Delta E_{MM}=\left(\Delta E_{vdW}+\Delta E_{ele}\right)\;$$

(4) $$\Delta G_{sol}=\Delta G_{GB}+\Delta G_{SA}$$

where Δ*G*_*complex*_ stands for the total binding free energy between the protein–ligand complexes. The total free energy of the protein and ligand in the solvent was expressed by Δ*G*_*protein*_ and Δ*G*_*ligand*_. In Equation (2), the Δ*E*_*MM*_ stands for the interaction energy between the protein–ligand complex under the gas-phase condition, which was estimated by calculating the van der Waals (Δ*E*_*vdW*_) and electrostatic (Δ*E*_*ele*_) energies (Equation (3)). The Δ*G*_*sol*_ stands for the free energy solvation, which was derived by calculating the polar solvation Δ*G*_*GB*_ and non-polar solvation Δ*G*_*SA*_ energy in Equation (4). The entropy contribution of the system is represented by *T*Δ*S*. The entropy (−*T*Δ*S*) calculation through nmode or Quasi-harmonic (QH) approximation is a computationally cumbersome process; therefore, the entropy term, (−*T*Δ*S*) was not considered in this study.

The final 2 ns trajectories from each system were taken for the MM-PB(GB)SA total binding energy and per-residue binding energy decomposition analysis.

For the end-state LIE calculation, each of the docked ligands was simulated in an unbound state explicitly by keeping the same parameters as described in the MD simulation method. In the LIE method, the binding affinity (ΔG_*bind*_) can be written as:

(5) $$\begin{array}{c} {\Delta G_{bind}=\alpha (\langle V_{lig-surr\;}^{vdW}\rangle }_{bound}-{\langle V_{lig-surr\;}^{vdW}\rangle }_{unbound})+\\ {\beta (\langle V_{lig-surr\;}^{ele}\rangle }_{bound}-{\langle V_{lig-surr\;}^{ele}\rangle }_{unbound}) \end{array}$$

The Coulomb interaction ($V_{lig-surr\;}^{vdW}$) and electrostatic interaction ($V_{lig-surr\;}^{ele}$) values in unbound form were computed by the ‘*gmx enemat*’ function. These values were supplied in the final LIE calculation using the ‘*gmx lie*’ function. As described in these studies [44,45,46], we used the scaling factors α = 0.181 and β = 0.43 by assigning the neutral charge to the ligands.

### 3.4. Dataset Building, Molecular Alignment, and CoMFA-CoMSIA (3D-QSAR) Study

The dataset of 40 pyrimidine-4,6-diamine-based compounds reported to be FLT3 inhibitors was taken for this study. We selected the last 1 ns MD average structure of compound M01 as a template structure. Based on the template structure, the rest of the compounds were sketched, and partial charges were assigned by the Gasteiger method in SYBYL-X2.1 (Tripos, Inc., St. Louis, MO, USA). All compounds were then subjected to minimization by tripos forcefield using the 0.05 kcal/mol convergence criterion with a maximum iteration of 2000 runs. Molecular alignment is an essential step towards the development of the 3D-QSAR model. We used the ‘common substructure-based alignment’ and ‘database alignment’ functions available in SYBYL-X2.1 to align compounds over the template structure as described in earlier studies [47,48].

For the 3D-QSAR study, the IC_50_ values of the compounds were converted into logarithmic IC_50_ (pIC_50_) values. The dataset was divided into the training set of 30 compounds to develop the model and a test set of 10 compounds to evaluate the external predictive capability. CoMFA and CoMSIA are the two widely established 3D-QSAR approaches, which were used to establish the structure–activity relationship of the compounds in the dataset. In CoMFA, the steric fields (S) were calculated using the Lennard–Jones potential function, whereas the electrostatic fields (E) were calculated using the Coulombic potential function. The compounds were placed one after another in a spatial grid box with a grid spacing of 2.0 by maintaining an energy tolerance of 30 kcal/mol. The *sp^3^* carbon atom was assigned as a probe by setting the van der Waals radii at 1.52 Å, with a net charge of +1.0. The other parameters were accepted by default in SYBYL-X2.1.

In the CoMSIA model, besides the steric and electrostatic fields, three additional descriptors, such as hydrophobic (H), H-bond acceptor (A), and H-bond donor (D) fields, were also adopted. The Gaussian-type functions were used to distinguish the distance between the probe atoms and the molecule’s atoms for all grid points. The rest of the parameters were kept similar to the CoMFA parameters. All descriptors were used in different combinations to get the best possible CoMSIA model.

The partial least squares (PLS) method was adopted to analyze the internal validation of CoMFA and CoMSIA models. The leave-one-out (LOO) method was applied to obtain the cross-validation coefficient (*q*^2^) and the optimal number of components (ONC) with a column-filtering value of 2.0 kcal/mol. Subsequently, the non-cross-validated correlation coefficient (*r*^2^), Fisher′s statistics (F value), and the standard error of estimation (SEE) were calculated, and finally, the pIC_50_ values were predicted for each compound of the test set. The value of *q*^2^ and *r*^2^ greater than 0.5 and 0.6, respectively, as well as *r*^2^−*q*^2^ not exceeding 0.3 well indicated the internal validity of the QSAR models.

The fitness of the CoMFA and CoMSIA models were assessed by estimating the chi-squared (χ^2^) and root-mean-squared error (RMSE) [29]:

(6) $$x^{2}=\sum_{i=1}^{n}\frac{{\left(y_{i}-{\hat{y}}_{i}\right)}^{2}}{y_{i}^{2}}$$

(7) $$\mathrm{RMSE}=\sqrt{\frac{\sum_{i=1}^{n}{\left(y_{i}-{\hat{y}}_{i}\right)}^{2}}{n-1}}$$

where *y_i_* and *ŷ_i_* are the observed and predicted activity, respectively, and n is the number of compounds being measured. The large χ^2^ (≥0.5) and RMSE (≥1.0) values reflect the poor predictive accuracy of the QSAR models.

The external validation of the CoMFA and CoMSIA by test set compounds was a crucial step in determining the true predictive power of any QSAR models. The following criteria proposed by Roy et al. [30], Gramatica et al. [31], and Todeschini et al. [32] were used to externally validate the models. The in-depth analysis process is described in the Supplementary Material under the Methodology Section. The progressive scrambling study was conducted as described in this study [49].

The applicability domain (AD) analysis of the QSAR models was done using the leverage approach as described in earlier studies [34]. The standardized residuals from the activity values of the training set and test set compounds were plotted against their leverage values in the Williams plot. The detailed method was described in the Supplementary Materials. The leverage value of any compound exceeding the warning leverage (h*), as shown with the red dotted line, was denoted as outliers and influenced the model quality.

### 3.5. Contour Maps Analysis

The contour maps were generated from CoMFA and CoMSIA to explain the structure–activity relationship of the existing compounds. The most active compound M01 was placed in the center as a reference, and the contour maps were shown as 3D StDev*Coeff to elucidate the field effects of the descriptors. The green and yellow contours signify the favorable and unfavorable substitutions for steric groups [50,51]. The blue and red contours convey the favorable and unfavorable substitutions for an electropositive group in CoMFA and CoMSIA. Similarly, the favorable and unfavorable substitutions for the hydrophobic, H-bond acceptor, and H-bond donor are represented by orange-gray, magenta-slate, and cyan-purple color schemes.

### 3.6. Designing of the New Compounds

Based on the SAR study, we designed 30 new compounds and predicted their activity by the CoMSIA (SEHD) model. Sixteen compounds out of them were predicted to be a higher pIC50 value than the most active compound M01. Thereafter, we analyzed physicochemical properties, the synthetic accessibility (SA) score by the SWISSADME [52] webserver, and the absorption, distribution, metabolism, excretion, and toxicity (ADMET) properties by the pkCSM [53] webserver of these compounds. Finally, the designed compounds were subjected to MM-PB(GB)SA binding free energy calculation.

## 4. Conclusions

The development of FLT3 inhibitors is a promising strategy for achieving the therapeutic goal of the treatment of AML. In this work, we used molecular docking and MD simulations to better understand the crucial interaction and stability of the ligands inside the binding pocket. The protein–ligand affinity was estimated by computing the MM-PB(GB)SA and LIE-binding energy, including the mutant FLT3 complexes. In a previously conducted docking study, residues Q575, L576, Q577, K644, F691, C694, L818, and L832 alongside the DFG motif (D829, F830, and G831) formed the fundamental docking interface for a group of diverse inhibitors. In our study, the combination of docking and MD study exposed several key residues, such as L616, M665, F691, E692, N701, L818, and F830 inside the ATP-binding pocket, which could be responsible for the affinity and selectivity of the type II inhibitors. K644 and C694 are the two important residues that form H-bond interactions. Subsequently, we established the CoMFA (*q*^2^ = 0.802, *r*^2^ = 0.983) and CoMSIA (*q*^2^ = 0.725, *r*^2^ = 0.965) models, both of which show a reasonable statistical correlation between actual and predictive activity and internal verification ability. The models also showed satisfactory stability and sensitivity in progressive scrambling analysis. The $Q_{F3}^{2}$ metrics from CoMFA and CoMSIA were found to be 0.698 and 0.668, respectively, indicating the external predictive power. The models could predict the activity values of new compounds having a similar scaffold. Following that, we developed the CoMFA and CoMSIA contour maps for the SAR study. Contour maps could provide crucial information regarding how chemical group substitution may improve the inhibitory activity of chemical compounds, which may be explained further by their docking and MD poses. Finally, we designed 30 new compounds, 16 of which had a higher predictive pIC_50_ than compound M01. In addition, free energy calculation of the selected designed compounds revealed a greater binding affinity to the FLT3. Compounds D02–D08, D12, D15, and D22, in particular, might offer potential inhibitory activity against FLT3.

In this study, several effective computational approaches and reliable statistics were used, and they may provide several key mechanistic interpretations at the molecular level. The overall findings of this work may be beneficial in delivering theoretical guidance in the future development and synthesis of *N*-phenylpyrimidine-4-amine-based FLT3 inhibitors.

## Figures and Tables

**Table 1:** MM-PB(GB)SA and LIE energy estimation of the FLT3 inhibitor complexes.

Complexes	MM-PB(GB)SA Binding Energy Terms in kcal/mol							LIE (kcal/mol)
	VDWAALS	E_EL_	E_PB/GB_	E_SURF_	ΔG_gas_	ΔG_solv_	ΔTOTAL	ΔG_bind_
**FLT3-M01**	−71.17	−51.49	68.69	−8.82	−122.66	59.86	**−62.80**	**−18.39**
**FLT3-M03**	−72.23	−275.18	295.32	−8.72	−347.42	286.60	**−60.27**	**−13.43**
**FLT3-M17**	−53.77	−21.27	34.20	−6.48	−75.04	27.72	**−47.32**	**−12.56**
**FLT3-M20**	−69.48	−222.37	240.20	−9.02	−291.85	231.17	**−60.68**	**−19.35**
**FLT3-M24**	−62.38	−28.98	39.64	−7.83	−91.36	31.80	**−59.56**	**−9.75**
**FLT3-M34**	−56.90	−248.23	262.59	−6.76	−305.14	255.82	**−49.31**	**−8.14**
**FLT3_(D835Y)_-M01**	−70.94	−51.33	67.31	−8.58	−122.28	58.72	**−63.55**	**−18.39**
**FLT3_(F691L)_-M01**	−69.99	−53.10	69.92	−8.86	−123.09	61.05	**−62.03**	**−17.95**
**FLT3_(D835Y,F691L)_-M01**	−70.43	−50.96	67.16	−8.56	−121.39	58.60	**−62.79**	**−17.95**

**VDWAALS**: van der Waals contribution from MM; **E_EL_**: electrostatic energy as calculated by the MM force field; **E_PB/GB_**: electrostatic contribution to the solvation free energy; **E_SURF_**: non-polar solvation free energy; **ΔG_gas_**: ΔG in gas phase; **ΔG_solv_**_:_ ΔG in solvation state; **ΔTOTAL**: total binding free energy from MM-PB(GB)SA; **ΔG_bind_**: final LIE binding energy; **LIE**: linear interaction energy.

**Table 2:** Per-residue MM-PB(GB)SA ΔTOTAL binding free energy decomposition in kcal/mol.

Residues	Compounds								
	M01	M03	M17	M20	M24	M34	M01 _(F691L)_	M01 _(D835Y)_	M01 _(F691L, D835Y)_
**K614**	NA	NA	NA	NA	NA	NA	−1.92	−0.66	−0.93
**L616**	−2.53	−1.90	−2.20	−2.20	−1.13	−2.44	−2.35	−2.38	−2.63
**V624**	NA	−1.61	−0.60	−1.50	−1.54	NA	−1.68	−1.60	−1.48
**A642**	−1.25	−1.26	NA	−1.04	−1.00	−1.05	−1.24	−1.30	−1.38
**K644**	−2.15	−2.26	0.36	−1.43	−0.75	−0.38	−1.84	−1.24	−2.24
**M665**	−1.48	−1.63	−0.96	−0.99	−1.39	−0.46	−1.46	−1.40	−1.42
**I674**	−1.03	−1.26	NA	−0.98	NA	NA	−0.92	−1.29	−0.91
**V675**	−1.81	NA	NA	−0.98	−1.64	−0.93	−1.69	−1.67	−1.62
**F691**	−2.44	−2.80	−3.63	−2.54	−2.76	−2.15	−1.40 *	−2.63	−1.09 *
**Y693**	−1.99	−2.04	−0.68	−1.73	−2.04	−1.38	−1.77	−1.98	−1.98
**C694**	−2.82	−2.24	−0.13	−0.74	−2.41	−0.77	−2.77	−2.72	−2.81
**G697**	−1.39	−0.77	−1.47	−1.29	−0.39	−1.72	−1.33	−1.41	−1.40
**L818**	−1.76	−1.67	−1.89	−1.67	−1.56	−1.70	−1.78	−1.68	−1.64
**C828**	−2.81	−2.86	NA	NA	−4.17	−0.96	−2.79	−3.27	−2.42
**D829**	−1.22	−1.41	−2.21	−1.12	−0.94	−2.08	−1.19	−1.29	−1.73
**F830**	−0.78	−2.35	−1.63	−1.71	−1.17	−1.61	NA	NA	NA

NA: Distance of the residues that are more than 4 Å from the compounds or contributed negligible binding energy to the ligand; (*): BE decomposition from the mutated residue.

**Table 3:** Statistics of the CoMFA and CoMSIA models.

Statistical Parameters	3D-QSAR (All Compounds)		3D-QSAR (Training Set Compounds)					Threshold Values
	CoMFA	CoMSIA (SEHA)	CoMFA	CoMSIA (SHD)	CoMSIA (SEHA)	CoMSIA (SEHD)	CoMSIA (SEHAD)	
***q* ^2^**	0.735	0.725	0.802	0.730	0.726	0.725	0.721	>0.5
**ONC**	6	5	6	5	5	5	5	
**SEP**	0.502	0.503	0.452	0.517	0.521	0.522	0.525	
***r* ^2^**	0.956	0.912	0.983	0.960	0.962	0.965	0.956	>0.6
**SEE**	0.204	0.284	0.134	0.199	0.194	0.186	0.209	<<1
**F-value**	119.97	70.84	216.62	114.54	121.34	131.90	104.16	>100
**χ^2^**	0.052	0.078	0.012	0.028	0.027	0.023	0.028	<0.5
**RMSE**	0.219	0.265	0.119	0.181	0.176	0.169	0.189	<0.3
**MAE**	<0.001	<0.001	<0.001	<0.001	<0.001	<0.001	<0.001	≈0
**RSS**	1.873	2.744	0.412	0.953	0.904	0.834	1.046	
***k***	NA	NA	1.033	1.022	1.032	1.033	1.037	0.85 ≤ *k* ≤ 1.15
***k*′**	NA	NA	0.962	0.969	0.962	0.961	0.958	0.85 ≤ *k*′ ≤ 1.15
***|r*_0_^2^*− r*′_0_^2^*|***	NA	NA	0.109	0.307	0.250	0.224	0.192	<0.3
**(*r*^2^ *− r*_0_^2^)/*r*^2^**	NA	NA	0.041	0.002	0.012	0.006	0.117	<0.1
***r_m_* ^2^**	NA	NA	0.724	0.603	0.635	0.649	0.664	>0.5
$\bar{r_{m}^{\;\;\;\;2}}$	NA	NA	0.694	0.449	0.492	0.511	0.623	>0.5
** Δ*r_m_* ^2^**	NA	NA	0.059	0.307	0.286	0.274	0.083	
$Q_{F3}^{2}$	NA	NA	0.698	0.604	0.656	0.668	0.660	>0.6
$Q_{ccc}^{2}$	NA	NA	0.821	0.746	0.778	0.787	0.787	
**S (%)**	52.9	20.2	54.4	28.9	18.5	20.5	16.2	
**E (%)**	47.1	29.9	45.6	NA	28.8	29.9	24.5	
**H%**	NA	37.0	NA	47.3	33.9	32.8	28.3	
**A%**	NA	13.0	NA	NA	18.8	NA	16.0	
**D%**	NA	NA	NA	23.8	NA	16.8	15.1	

***q*^2^**: squared cross-validated correlation coefficient; **ONC**: optimal number of components; **SEP**: standard error of prediction; ***r*^2^**: squared correlation coefficient; **SEE:** standard error of estimation; **F-value**: F-test value; **RMSE**: root-mean-squared error; **MAE**: mean absolute error; **RSS**: residual sum of error; ***k***: slope of the predicted vs. observed activity at zero intercept; ***k*′**: slope of the observed vs. predicted activity at zero intercept; ***r*_0_^2^**: squared correlation coefficient between predicted and observed activity; ***r*′_0_^2^**: squared correlation coefficient between predicted and observed activity; $Q_{F3}^{2}$: Q^2^ metrics for external test set validation; $Q_{ccc}^{2}$: concordance correlation coefficient; **S**: steric; **E**: electrostatic; **H**: hydrophobic; **A**: H-bond acceptor; **D**: H-bond donor; NA: Not applicable.

**Table 4:** Progressive scrambling results from the CoMFA and CoMSIA(SEHD) training set compounds.

Components	CoMFA			CoMSIA (SEHD)		
	*Q* ^2^	cSDEP	d*q*^2^/d*r*^2^*_yy_*_′_	*Q* ^2^	cSDEP	d*q*^2^/d*r*^2^*_yy_*_′_
1	0.191	0.828	0.175	0.230	0.807	0.306
2	0.358	0.750	0.731	0.429	0.707	0.996
3	0.477	0.689	0.855	0.491	0.662	1.052
4	0.489	0.696	1.480	0.550	0.652	1.221
5	0.479	0.711	1.821	**0.518**	**0.770**	**0.982**
6	**0.502**	**0.713**	**1.198**	0.410	0.709	1.513
7	0.518	0.718	1.663	0.400	0.796	2.285

**Table 5:** MM-PB(GB)SA binding energy estimation between FLT3 and newly designed compounds.

Complexes	MM-PB(GB)SA Binding Energy Terms in kcal/mol						
	VDWAALS	E_EL_	E_PB/GB_	E_SURF_	ΔG_gas_	ΔG_solv_	ΔTOTAL
**FLT3-D01**	−71.01	−45.14	66.81	−8.84	−116.16	57.96	**−58.19**
**FLT3-D02**	−70.82	−32.90	49.71	−9.17	−103.73	40.53	**−63.19**
**FLT3-D03**	−71.38	−55.60	71.34	−9.04	−126.98	62.30	**−64.68**
**FLT3-D04**	−72.37	−54.24	71.68	−9.30	−126.62	62.38	**−64.24**
**FLT3-D05**	−76.94	−63.43	79.81	−9.90	−140.38	69.90	**−70.47**
**FLT3-D07**	−73.59	−48.56	60.95	−9.45	−122.15	51.49	**−70.66**
**FLT3-D08**	−72.79	−33.30	50.01	−8.72	−106.10	41.29	**−64.81**
**FLT3-D09**	−74.03	−54.80	78.07	−9.34	−128.84	68.73	**−60.10**
**FLT3-D10**	−69.67	−49.27	71.01	−9.41	−118.94	61.59	**−57.35**
**FLT3-D11**	−80.40	−37.05	71.92	−10.24	−117.45	61.68	**−55.77**
**FLT3-D12**	−76.69	−27.84	46.29	−9.39	−104.53	36.90	**−67.63**
**FLT3-D14**	−80.42	−28.40	56.25	−10.07	−108.82	46.17	**−62.65**
**FLT3-D15**	−78.07	−57.04	70.96	−9.45	−135.11	61.50	**−73.61**
**FLT3-D17**	−66.50	−64.74	81.42	−8.66	−131.24	72.75	**−58.49**
**FLT3-D21**	−72.32	−17.78	46.93	−9.08	−90.11	37.84	**−52.27**
**FLT3-D22**	−73.23	−25.85	45.39	−9.44	−79.08	35.94	**−63.14**

**VDWAALS**: van der Waals contribution from MM; **E_EL_**: electrostatic energy as calculated by the MM force field; **E_PB/GB_**: electrostatic contribution to the solvation free energy; **E_SURF_**: non-polar solvation free energy; **ΔG_gas_**: ΔG in gas phase; **ΔG_solv_**_:_ ΔG in solvation state; **ΔTOTAL**: total binding free energy from MM-PB(GB)SA.

## Data Availability

The authors declared the availability of all materials and data upon request.

## Supplementary Materials

The following are available online at https://www.mdpi.com/article/10.3390/ijms222212511/s1.

## Abbreviations

FLT3	FMS-like tyrosine kinase-3
AML	acute myeloid leukemia
MM-PB(GB)SA	Molecular Mechanics Poison–Boltzmann/Generalized Born Surface Area
LIE	linear interaction energy
BE	binding Energy
CoMFA	comparative molecular field analysis
CoMSIA	comparative molecular similarity indices analysis
3D-QSAR	three-dimensional structure–activity relationship
