# Supplementary material for: Molecular Modeling Studies of N-phenylpyrimidine-4-amine Derivatives for Inhibiting FMS-like Tyrosine Kinase-3
Source: Int J Mol Sci. 2021 Nov 19;22(22):12511. doi: 10.3390/ijms222212511 (PMC8622510; doi:10.3390/ijms222212511)
Supplement: Supplementary file 1 [file ijms-22-12511-s001.zip › ijms-1448071-supplementary.pdf]

Supplementary Data: Molecular modeling studies of *N*-phenylpyrimidine-4-amine derivatives for inhibiting FMS-like tyrosine kinase-3

Suparna Ghosh [1], Seketoulie Keretsu [1], Seung Joo Cho [1,2,]✉

[1]Department of Biomedical Sciences, College of Medicine, Chosun University, Gwangju 501-759, Republic of Korea; s.ghosh@chosun.kr (S.G); keretsu@chosun.kr (S.K); chosj@chosun.ac.kr (S.J.C)

[2]Department of Cellular and Molecular Medicine, College of Medicine, Chosun University, Gwangju 501-759, Republic of Korea

✉Correspondence: chosj@chosun.ac.kr; Tel.: +82-62-230-7482 (office) or +82-11-479-1010 (cell phone)

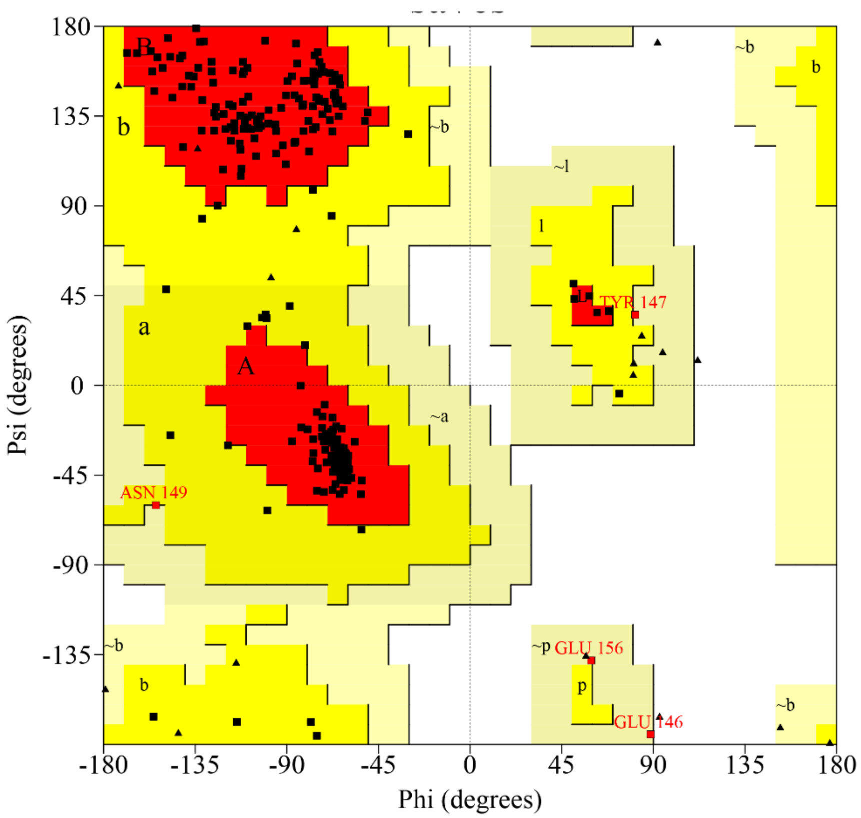

Supplementary Figure S1: Ramachandran plot analysis of the loop modeled FLT3 structure. After modeling the missing residues and loops fall within the well-accepted regions.

Supplementary Table S1. ligRMSD evaluations of the docked compounds

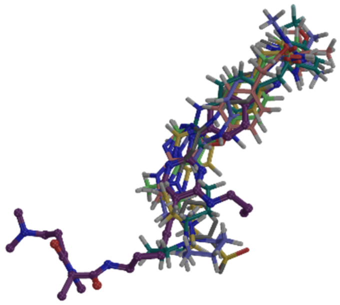

Reference molecule: FF-10101 (PDB ID: 5X02) in ball-stick representation.

Molecules	Match Type	RMSD (Å)	% ref match	% molecule match	ΔG (Kcal/mol)
**M01**	strict	3.04	10.26	10.53	-12.31
**M03**	strict	2.45	28.21	29.73	-12.68
**M17**	flexible	2.58	43.59	65.38	-9.88
**M20**	strict	2.31	28.21	29.73	-10.54
**M24**	flexible	2.68	43.59	58.62	-9.68
**M34**	strict	2.42	28.21	40.74	-10.09

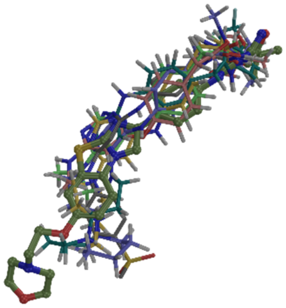

Reference molecule: Quizartinib (PDB ID: 4XUF) in ball-stick representation.

Molecules	Match Type	RMSD (Å)	% ref match	% molecule match
**M01**	flexible	2.25	47.50	50.00
**M03**	flexible	1.90	47.50	51.35
**M17**	flexible	1.86	40.00	61.54
**M20**	strict	5.24	22.50	24.32
**M24**	strict	1.40	12.50	17.24
**M34**	flexible	1.82	27.50	40.74

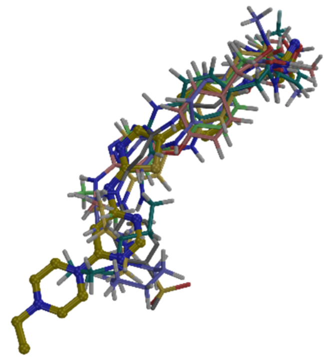

Reference molecule: AWO (PDB ID: 6ITT) in ball-stick representation.

Molecules	Match Type	RMSD (Å)	% ref match	% molecule match
**M01**	flexible	1.76	45.95	44.74
**M03**	flexible	1.66	45.95	45.95
**M17**	flexible	1.74	43.24	61.54
**M20**	flexible	1.77	29.73	29.73
**M24**	strict	0.98	8.11	10.34
**M34**	strict	4.91	24.32	33.33

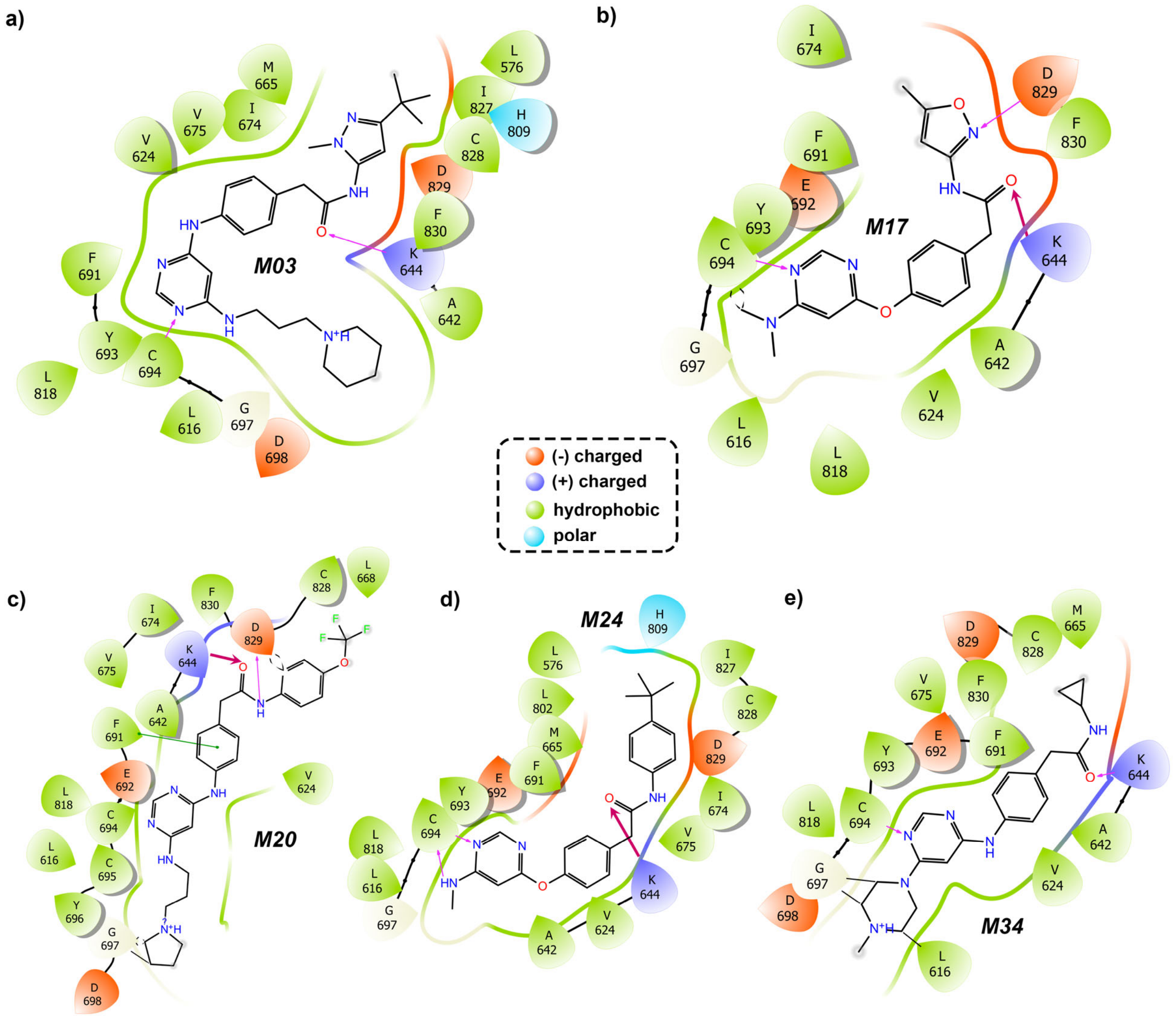

Supplementary Figure S2. Molecular Docking analysis of compound M03, M17, M20, M24, and M34 in 2D illustration. Residues were shown by distinct color scheme based on their chemical properties. H-bonds were shown by magenta arrows.

Supplementary Table S2. Structure and activity values of N-methylpyrimidine-4-amine based FLT3 inhibitors

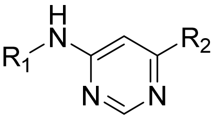

Structure A

#Cpd	Structure	R_1	R_2	pIC_{50}
01	A	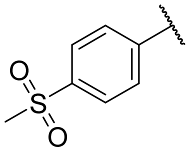	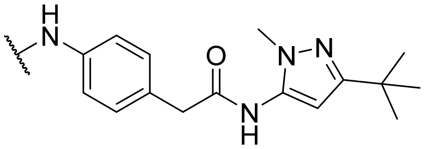	7.85
02	A	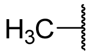	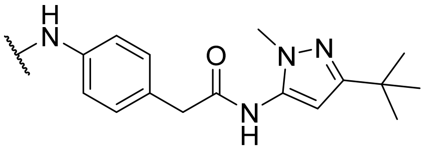	7.42
03	A	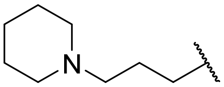	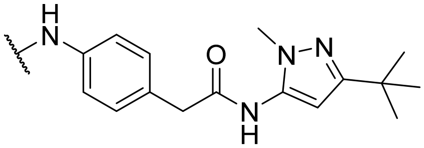	6.88
04	A	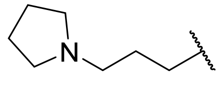	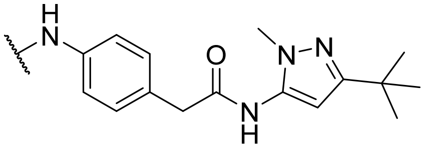	6.42
05	A	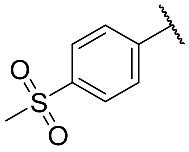	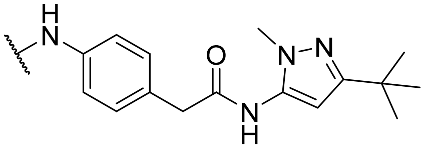	6.57
06	A	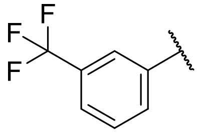	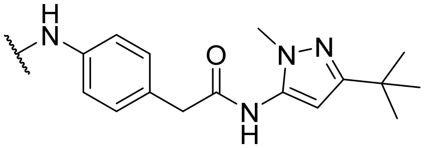	6.01
07	A	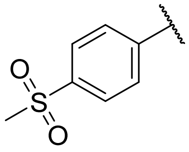	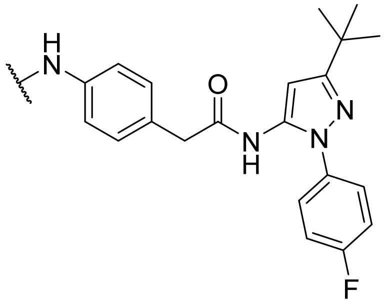	5.49

08	A	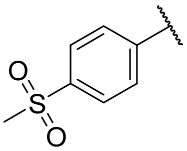	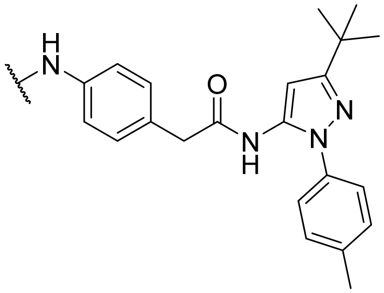	5.31
09		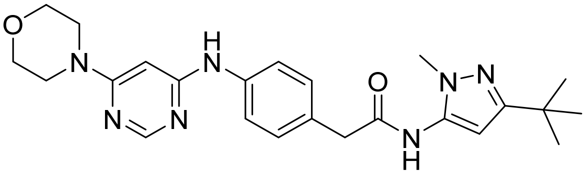		6.36
10		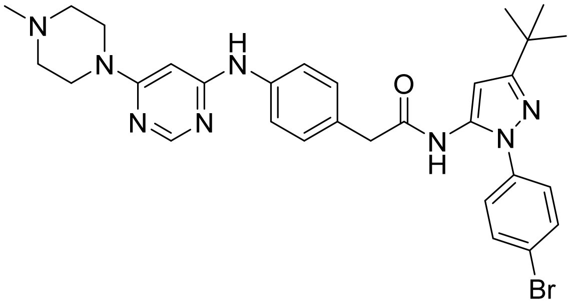		5.22
11*	A	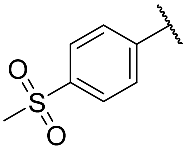	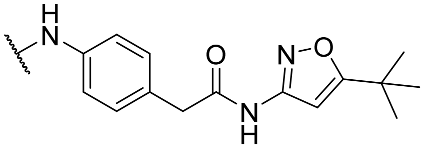	7.20
12	A	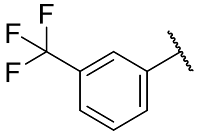	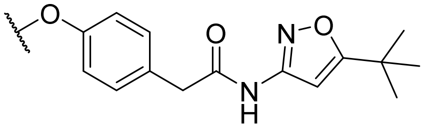	6.24
13	A	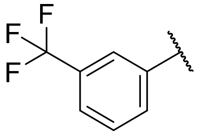	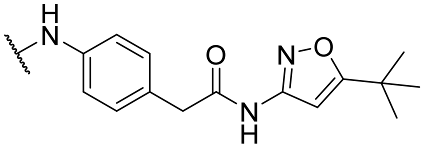	5.92
14	A	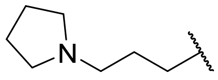	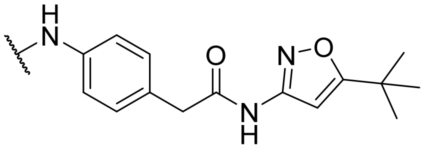	5.98
15		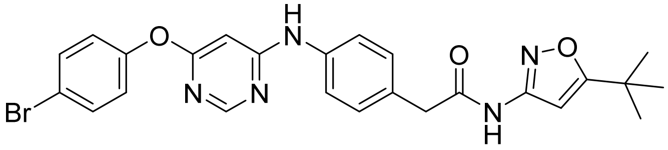		4.82
16		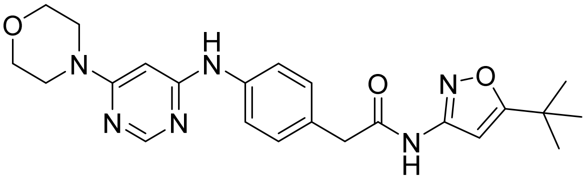		6.40

17*		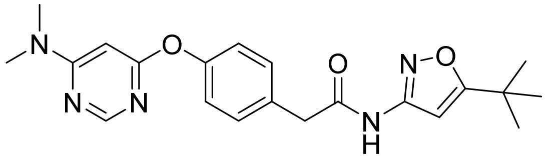		5.05
18*	A	H_3C—	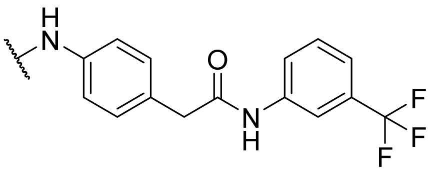	6.38
19	A	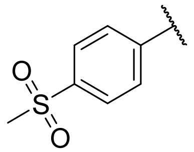	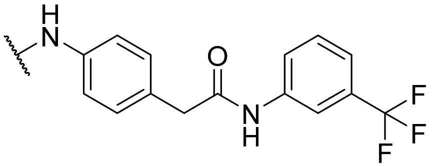	6.06
20*	A	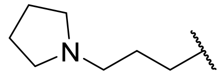	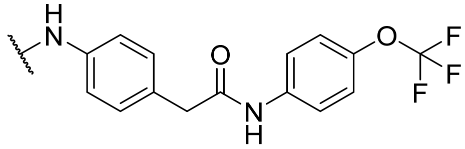	6.04
21*	A	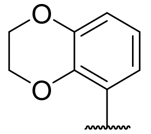	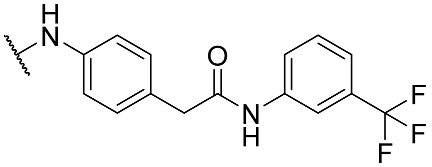	5.87
22	A	H_3C—	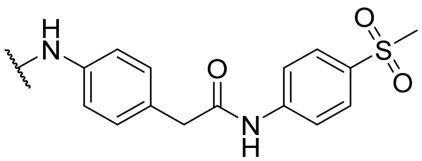	5.67
23*	A	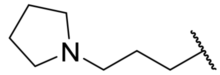	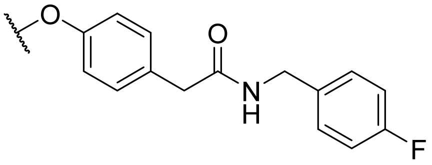	5.34
24*	A	H_3C—	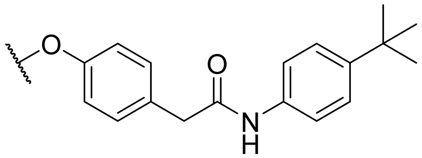	5.29
25	A	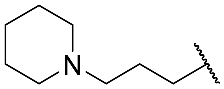	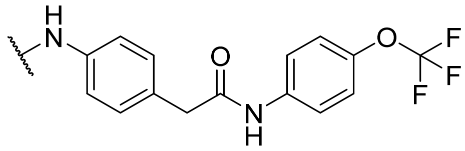	5.18
26*	A	H_3C—	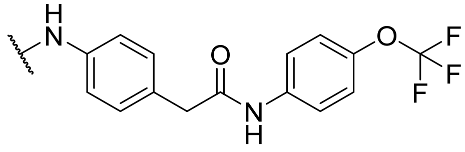	5.13
27	A	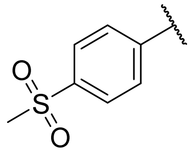	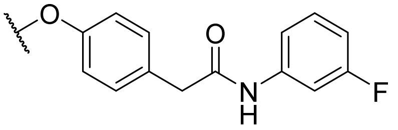	5.10

28	A	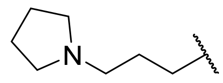	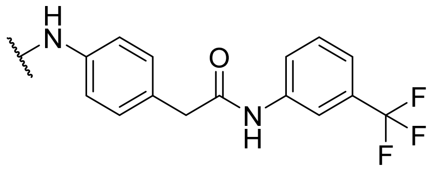	5.03
29*	A	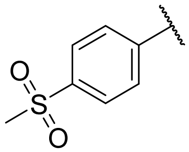	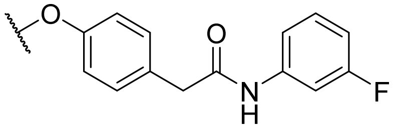	4.93
30	A	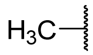	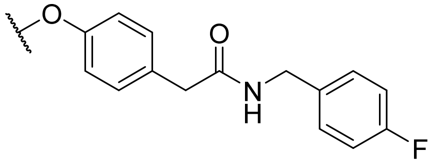	4.89
31	A	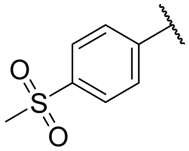	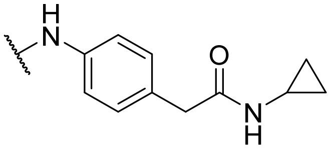	5.25
32	A	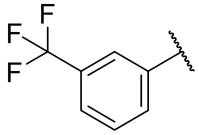	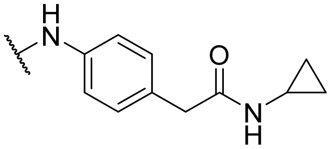	5.09
33	A	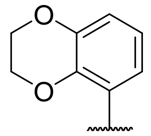	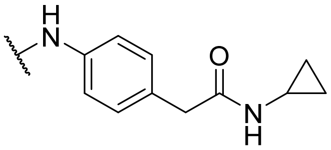	4.97
34		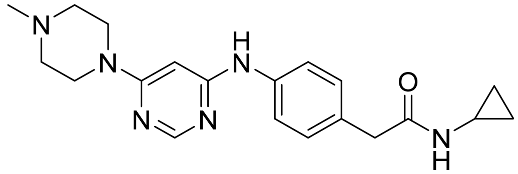		4.86
35		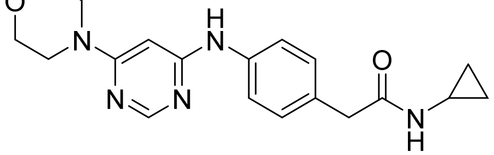		4.82
36	A	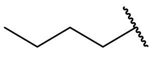	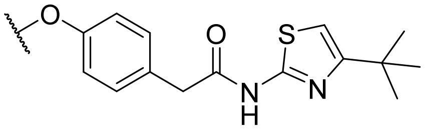	5.12
37	A	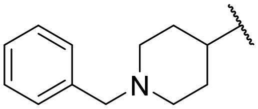	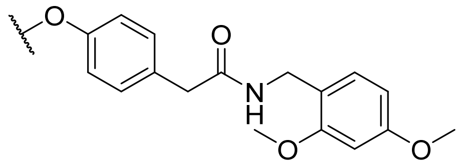	4.86
38	A	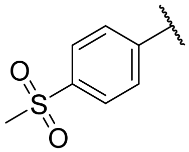	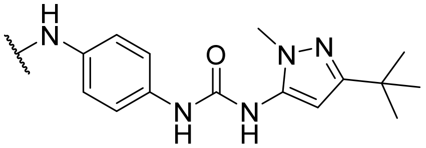	7.38

39	A	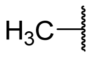	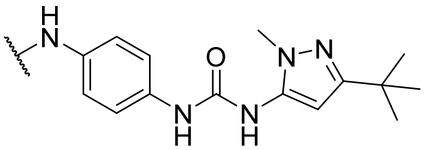	7.41
40*	A	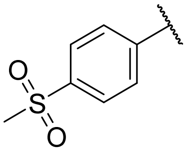	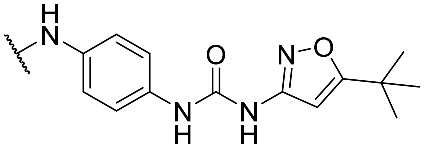	7.53

(*) Test set compounds. #**Cpd.**: Compounds

Supplementary Table S3. Detailed statistical values of the CoMSIA models (all compounds)

CoMSIA	q^2	ONC	SEP	r^2	SEE	F-value	Field Contribution				
							S	E	H	A	D
**S**	0.656	6	0.571	0.876	0.342	38.99	100	-	-	-	-
**E**	0.507	4	0.664	0.775	0.449	30.07	-	100	-	-	-
**H**	0.684	4	0.531	0.875	0.334	61.27	-	-	100	-	-
**A**	0.330	5	0.786	0.619	0.592	11.05	-	-	-	100	-
**D**	0.235	3	0.816	0.438	0.699	9.36	-	-	-	-	100
**SE**	0.701	6	0.533	0.924	0.269	66.89	47.1	52.9	-	-	-
**EH**	0.708	4	0.511	0.899	0.301	77.68	-	44.0	56.0	-	-
**EA**	0.496	6	0.691	0.822	0.412	25.32	-	73.5	-	26.5	-
**ED**	0.510	4	0.662	0.773	0.451	29.77	-	59.5	-	-	40.5
**SH**	0.702	4	0.516	0.881	0.326	64.81	34.6	-	65.4	-	-
**SA**	0.670	6	0.559	0.926	0.266	68.35	62.9	-	-	37.1	-
**SD**	0.554	6	0.651	0.856	0.369	32.73	68.5	-	-	-	31.5
**HA**	0.703	5	0.523	0.897	0.308	59.27	-	-	71.5	28.5	-
**HD**	0.634	5	0.581	0.904	0.297	64.22	-	-	73.5	-	26.5
**AD**	0.356	5	0.770	0.721	0.507	17.54	-	-	-	46.5	53.5
**SHE**	0.722	5	0.506	0.917	0.277	74.76	23.9	34.9	41.2	-	-
**SEA**	0.678	6	0.552	0.915	0.284	59.31	38.9	45.3	-	15.8	-
**SED**	0.647	6	0.579	0.933	0.252	76.72	34.2	42.8	-	-	23.0
**EHA**	0.710	5	0.517	0.911	0.286	69.67	-	35.9	47.7	16.4	-
**EHD**	0.674	5	0.548	0.917	0.277	74.76	-	34.0	42.8	-	23.2
**SHA**	0.718	5	0.510	0.902	0.300	62.58	27.2	-	51.7	21.1	-
**SHD**	0.645	5	0.572	0.894	0.312	57.47	28.0	-	50.7	-	21.4
**EAD**	0.493	4	0.673	0.764	0.460	28.30	-	46.0	-	19.8	34.2
**HAD**	0.610	5	0.600	0.830	0.390	42.73	-	-	53.5	19.9	26.6
**SEHD**	0.682	5	0.542	0.922	0.269	79.96	19.6	28.4	33.9	-	18.1
**SEHA**	0.725	5	0.503	0.912	0.284	70.84	20.2	29.9	37.0	13.0	-

**SEAD**	0.587	6	0.626	0.895	0.316	46.76	29.3	35.0	-	11.3	24.4
**EHAD**	0.656	5	0.563	0.890	0.319	54.83	-	29.1	37.4	10.5	22.9
**SHAD**	0.573	5	0.573	0.882	0.330	50.86	22.6	-	41.3	15.1	21.1
**SEHAD**	0.676	5	0.546	0.895	0.311	57.75	16.9	24.5	30.7	9.1	18.8

q^2: squared cross-validated correlation coefficient; **ONC**: optimal number of components; **SEP**: standard error of prediction; r^2: squared correlation coefficient; **SEE**: standard error of estimation; **F-value**: F-test value; r^2_{pred}: predictive r^2; **S**: Steric; **E**: Electrostatic; **H**: Hydrophobic; **A**: H-bond acceptor; **D**: H-bond donor. Green highlighted box showing the final selection of CoMSIA model.

Supplementary Table S4. Detailed statistical value of the CoMSIA models (Training Set compounds)

CoMSIA	q^2	ONC	SEP	r^2	SEE	F-value	Field Contribution				
							S	E	H	A	D
**S**	0.568	6	0.668	0.946	0.236	67.08	100	-	-	-	-
**E**	0.490	3	0.683	0.847	0.375	47.31	-	100	-	-	-
**H**	0.659	3	0.558	0.918	0.274	96.80	-	-	100	-	-
**A**	0.322	3	0.787	0.637	0.575	15.23	-	-	-	100	-
**D**	0.219	4	0.861	0.583	0.629	8.74	-	-	-	-	100
**SE**	0.619	5	0.614	0.960	0.199	115.25	40	60	-	-	-
**EH**	0.691	4	0.542	0.955	0.207	132.95	-	47.6	52.4	-	-
**EA**	0.495	4	0.693	0.844	0.385	33.79	-	61.5	-	38.5	-
**ED**	0.516	4	0.678	0.873	0.347	43.11	-	64.6	-	-	35.4
**SH**	0.677	4	0.554	*0.946*	0.227	109.31	35.8	-	64.2	-	-
**SA**	0.644	6	0.607	0.958	0.209	87.06	56.4	-	-	43.6	-
**SD**	0.688	6	0.568	0.941	0.247	60.83	63.2	-	-	-	36.8
**HA**	0.700	5	0.545	0.939	0.245	74.49	-	-	63.1	36.9	-
**HD**	0.684	4	0.548	0.940	0.239	97.82	-	-	69.5	-	30.5
**AD**	0.429	3	0.722	0.791	0.437	32.77	-	-	-	59.5	40.5
**SHE**	0.714	3	0.511	0.946	0.223	150.47	23.2	36.4	40.4	-	-
**SEA**	0.644	6	0.606	0.965	0.190	105.55	31.4	44.4	-	24.2	-
**SED**	0.632	6	0.617	0.966	0.186	110.17	33.6	45.0	-	-	21.4
**EHA**	0.711	6	0.546	0.967	0.184	113.19	-	35.6	42.8	21.6	-
**EHD**	0.687	5	0.556	0.960	0.198	115.98	-	37.8	41.8	-	20.4
**SHA**	0.719	5	0.527	0.953	0.215	98.13	24.8	-	46.6	28.7	-
**SHD**	0.730	5	0.517	0.960	0.199	114.54	28.9	-	47.3	-	23.8
**EAD**	0.512	3	0.668	0.829	0.395	41.98	-	44.8	-	29.6	25.6
**HAD**	0.667	5	0.574	0.935	0.253	69.46	-	-	48.5	28.8	22.7
**SEHD**	0.725	5	0.522	0.965	0.186	131.90	20.5	29.9	32.8	-	16.8
**SEHA**	0.726	5	0.521	0.962	0.194	121.34	18.5	28.8	33.9	18.8	-

**SEAD**	0.619	5	0.614	0.931	0.261	65.01	23.5	33.5	-	20.4	22.6
**EHAD**	0.687	6	0.569	0.959	0.207	88.53	-	29.8	35.6	18.0	16.5
**SHAD**	0.706	5	0.539	0.948	0.226	88.10	20.9	-	37.3	22.6	19.3
**SEHAD**	0.721	5	0.525	0.956	0.209	104.16	16.2	24.5	28.3	16.0	15.1

q^2: squared cross-validated correlation coefficient; **ONC**: optimal number of components; **SEP**: standard error of prediction; r^2: squared correlation coefficient; **SEE**: standard error of estimation; **F-value**: F-test value; r^2_{pred}: predictive r^2; **S**: Steric; **E**: Electrostatic; **H**: Hydrophobic; **A**: H-bond acceptor; **D**: H-bond donor. Yellow-orange highlighted box were selected for **Table-2**.

Supplementary Table S5. Actual pIC_{50} vs predicted pIC_{50} values with their residuals of selected CoMFA and CoMSIA models

#Cpd	Actual pIC_{50}	3D-QSAR (All Compounds)				3D-QSAR (Training Set Compounds)									
		CoMFA		CoMSIA (SEHA)		CoMFA		CoMSIA (SHD)		CoMSIA (SEHA)		CoMSIA (SEHD)		CoMSIA (SEHAD)	
		Pred	Resi	Pred	Resi	Pred	Resi	Pred	Resi	Pred	Resi	Pred	Resi	Pred	Resi
**01**	7.85	7.18	0.67	7.12	0.73	4.87	-0.05	7.90	-0.04	7.88	-0.03	7.97	-0.11	7.90	-0.04
**02**	7.42	7.41	0.01	7.17	0.25	4.80	0.02	7.03	0.39	7.09	0.33	7.08	0.34	6.93	0.48
**03**	6.88	6.82	0.06	6.79	0.09	4.78	0.07	6.57	0.31	6.57	0.31	6.47	0.41	6.52	0.36
**04**	6.42	6.80	-0.37	6.77	-0.35	4.98	-0.11	6.53	-0.11	6.56	-0.14	6.48	-0.05	6.52	-0.10
**05**	6.57	6.25	0.31	6.51	0.05	4.87	0.01	6.67	-0.10	6.66	-0.09	6.58	-0.01	6.69	-0.12
**06**	6.01	6.17	-0.15	6.13	-0.11	5.48	-0.55	6.06	-0.04	6.02	-0.00	6.08	-0.06	6.06	-0.05
**07**	5.49	5.53	-0.04	5.82	-0.33	5.03	-0.06	5.66	-0.16	5.72	-0.23	5.67	-0.18	5.72	-0.23
**08**	5.31	5.46	-0.15	5.59	-0.27	5.18	-0.14	5.54	-0.23	5.55	-0.23	5.51	-0.20	5.57	-0.26
**09**	6.36	6.43	-0.06	6.40	-0.03	4.98	0.07	6.46	-0.10	6.62	-0.25	6.61	-0.24	6.50	-0.13
**10**	5.22	4.99	0.22	4.70	0.51	5.04	0.04	4.92	0.30	4.90	0.31	5.03	0.18	4.98	0.23
**11***	7.20	7.08	0.11	6.90	0.30	5.11	-0.00	6.03	1.16	6.36	0.83	6.28	0.92	6.28	0.91
**12**	6.24	6.11	0.13	6.19	0.05	5.08	0.03	6.39	-0.15	6.13	0.10	6.25	-0.00	6.21	0.02
**13**	5.92	5.90	0.01	5.72	0.20	5.70	-0.56	5.96	-0.03	6.03	-0.11	5.93	-0.00	5.99	-0.06
**14**	5.98	5.99	-0.01	5.86	0.11	5.00	0.18	5.86	0.11	6.01	-0.02	5.90	0.08	5.96	0.01
**15**	4.82	4.96	-0.13	5.02	-0.19	4.95	0.26	4.72	0.10	4.88	-0.05	4.72	0.10	4.83	0.00
**16**	6.40	6.49	-0.09	6.47	-0.06	5.32	-0.06	6.36	0.03	6.28	0.11	6.35	0.04	6.23	0.17
**17***	5.05	5.02	0.03	5.12	-0.06	4.90	0.39	5.15	-0.09	5.23	-0.18	5.07	-0.01	5.10	-0.04
**18***	6.38	6.01	0.37	5.88	0.50	5.49	-0.18	5.77	0.61	5.80	0.58	5.77	0.61	5.63	0.75
**19**	6.06	5.98	0.08	5.83	0.23	5.02	0.31	5.71	0.35	5.72	0.33	5.72	0.34	5.67	0.38
**20***	6.04	6.19	-0.15	6.12	-0.07	5.56	-0.07	6.48	-0.43	6.19	-0.15	6.20	-0.16	6.14	-0.10
**21***	5.87	5.59	0.28	5.51	0.36	5.75	-0.08	5.24	0.63	5.05	0.82	5.19	0.68	5.09	0.78
**22**	5.67	5.62	0.04	5.67	-0.00	5.21	0.66	5.69	-0.01	5.70	-0.03	5.70	-0.03	5.70	-0.02
**23***	5.34	5.14	0.19	5.27	0.07	5.99	-0.07	5.69	-0.35	5.22	0.12	5.24	0.09	5.33	0.00
**24***	5.29	5.17	0.12	5.16	0.13	6.06	-0.07	5.04	0.25	5.07	0.21	5.04	0.24	5.06	0.23
**25**	5.18	4.93	0.25	5.08	0.10	6.09	-0.07	5.13	0.05	4.99	0.19	5.05	0.13	5.07	0.11
**26***	5.13	5.55	-0.42	5.51	-0.37	6.10	-0.06	5.63	-0.50	5.52	-0.39	5.71	-0.57	5.54	-0.41
**27**	5.10	5.08	0.01	5.07	0.03	5.85	0.21	4.93	0.17	5.05	0.04	5.01	0.08	5.04	0.06
**28**	5.03	5.33	-0.29	5.42	-0.39	6.16	0.08	5.42	-0.38	5.26	-0.22	5.31	-0.27	5.34	-0.30
**29***	4.93	5.34	-0.41	5.34	-0.41	6.42	-0.05	5.25	-0.32	5.52	-0.59	5.36	-0.43	5.37	-0.44
**30**	4.89	5.10	-0.21	5.10	-0.21	5.81	0.56	5.07	-0.18	5.11	-0.22	5.07	-0.18	5.09	-0.20
**31**	5.25	5.11	0.14	5.33	-0.08	6.47	-0.07	5.24	0.00	5.40	-0.15	5.31	-0.06	5.38	-0.12
**32**	5.09	5.02	0.06	5.15	-0.05	6.54	-0.12	5.13	-0.04	5.02	0.06	5.10	-0.01	5.08	0.00
**33**	4.97	5.20	-0.23	5.32	-0.34	6.65	-0.08	5.12	-0.14	5.03	-0.05	5.04	-0.07	5.06	-0.09
**34**	4.86	4.85	0.01	4.75	0.11	6.66	0.22	4.93	-0.06	4.95	-0.08	4.94	-0.07	4.93	-0.06
**35**	4.82	4.84	-0.02	4.64	0.18	6.48	0.71	4.94	-0.11	4.86	-0.04	4.92	-0.10	4.86	-0.04
**36**	5.12	5.06	0.05	5.09	0.02	7.24	0.14	4.97	0.14	4.91	0.20	4.99	0.12	4.93	0.18

**37**	4.86	4.85	0.00	4.89	-0.02	7.48	-0.06	4.81	0.05	4.81	0.05	4.87	-0.01	4.78	0.08
**38**	7.38	7.49	-0.11	7.64	-0.2	7.26	0.15	7.31	0.06	7.39	-0.00	7.35	0.02	7.47	-0.08
**39**	7.41	7.64	-0.22	7.67	-0.26	7.19	0.34	7.58	-0.16	7.47	-0.05	7.59	-0.18	7.60	-0.18
**40***	7.53	7.66	-0.12	7.67	-0.13	7.90	-0.05	7.20	0.33	7.04	0.49	7.08	0.45	7.18	0.35

(*) Test set compound. **Pred:** Predicted IC_{50}; **Resi**: Residual; **S**: steric; **E**: electrostatic; **H**: Hydrophobic; **A**: H-bond acceptor; **D**: H-bond donor. **Green highlight**: Outliers (difference of the predicted value is over 1 log unit from the actual value).

Supplementary Table S6. Designing of the New Compounds and their predicted pIC_{50} values

Compounds	Structure	Predicted pIC_{50}
*D01	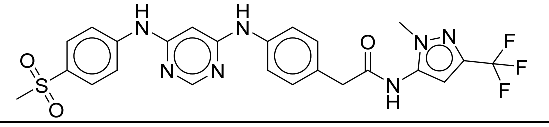	7.90
*D02	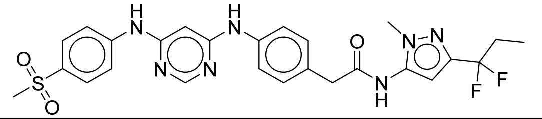	7.93
*D03	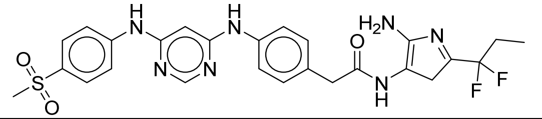	7.92
*D04	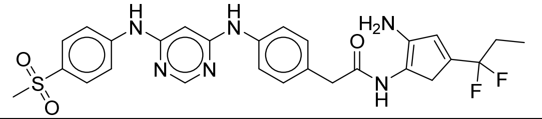	7.93
*D05	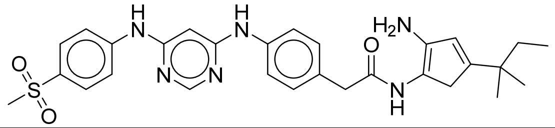	7.86
D06	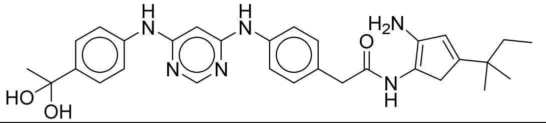	6.97
*D07	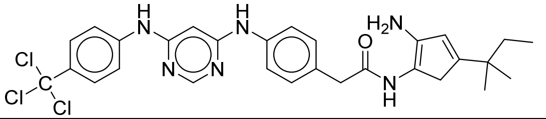	7.84
*D08	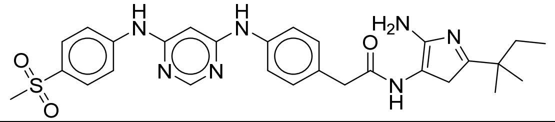	7.84
*D09	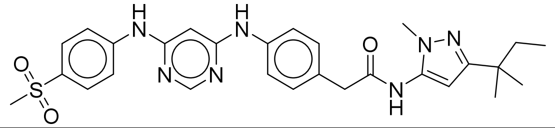	7.91
*D10	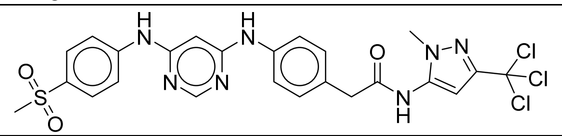	8.10

*D11	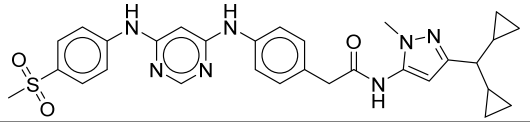	7.80
*D12	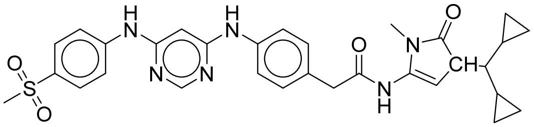	7.86
D13	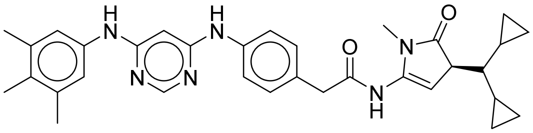	6.94
*D14	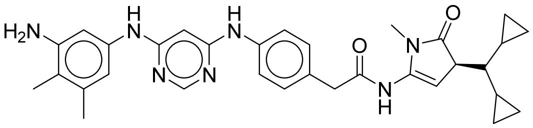	7.87
*D15	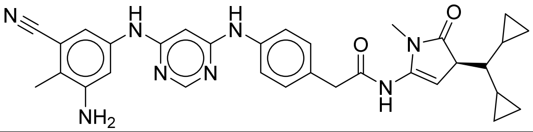	8.00
D16	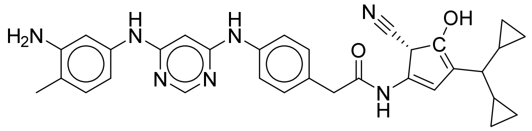	5.93
*D17	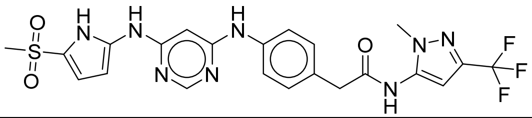	7.89
D18	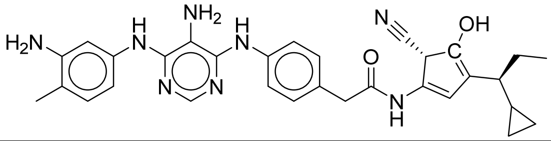	6.41
D19	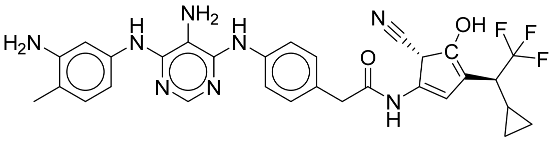	6.25
D20	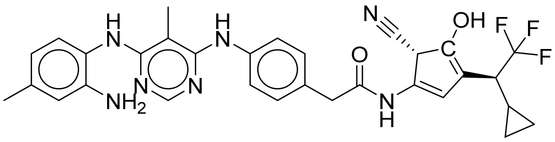	6.12
*D21	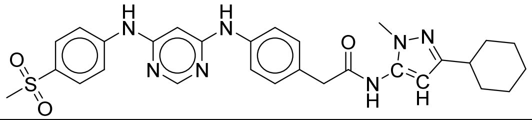	9.43
*D22	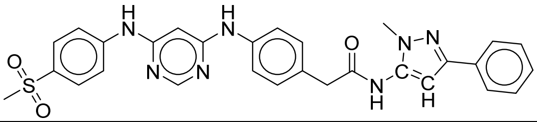	9.30
D23	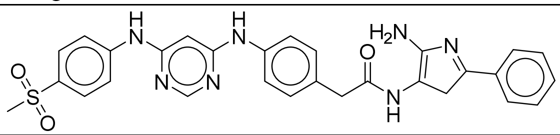	7.59

D24	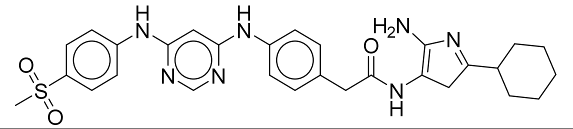	7.72
D25	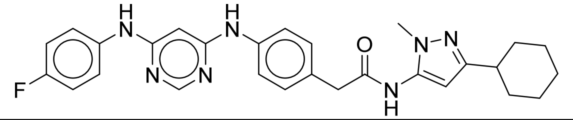	7.45
D26	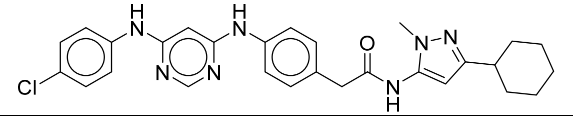	7.33
D27	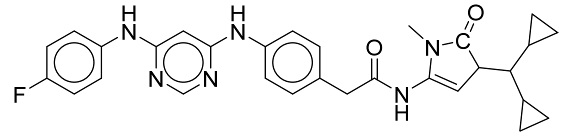	7.07
D28	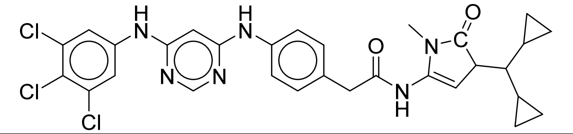	7.02
D29	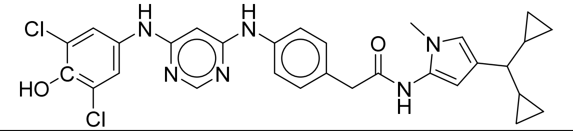	7.35
D30	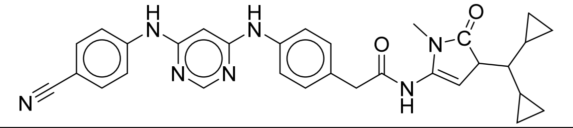	7.08

D: Designed

Supplementary Table S7: Predicted pIC_{50}, Docking Score, Physicochemical property and Synthetic Accessibility score prediction

Compounds	Pred. pIC_{50}	Docking Score (ΔG in Kcal/mol)	xlogP value	H-bond donor	H-bond acceptor	Molecular Mass (MM)	Rotatable bonds	TPSA (Å)	PAINS	SA
D01	7.90	-12.35	3.42	3	4	545.14	9	139.28	Pass	3.56
D02	7.93	-11.86	3.66	3	4	555.18	10	139.28	Pass	3.89
D03	7.92	-11.97	1.61	4	6	555.18	10	159.84	Pass	4.52
D04	7.93	-10.63	2.31	4	5	554.19	10	147.48	Pass	4.42
D05	7.86	-10.20	3.75	6	7	528.28	10	147.48	Pass	4.57
D07	7.84	-10.52	6.42	4	5	584.16	10	147.48	Pass	4.57
D08	7.84	-10.39	3.59	3	6	547.23	10	159.84	Pass	4.63
D09	7.91	-10.93	2.62	3	6	545.14	9	139.28	Pass	4.07
D10	8.10	-11.43	2.98	3	6	545.14	9	139.28	Pass	3.56
D11	7.80	-10.78	4.30	3	6	571.23	11	139.28	Pass	4.17
D12	7.86	-11.53	5.12	4	5	550.29	10	125.27	Pass	4.82
D14	7.98	-10.90	5.31	4	5	551.30	10	125.27	Pass	5.05
D15	8.00	-11.64	4.14	4	6	561.27	10	149.06	Pass	4.77
D17	7.89	-11.02	3.05	4	7	534.14	9	155.07	Pass	3.63
D21	9.43	-9.47	4.19	3	8	559.23	9	137.06	Pass	4.61

D22	9.30	-9.81	3.99	3	8	553.18	9	137.06	Pass	4.86

Supplementary Table S8: In silico ADMET assessment of the designed compounds

Compounds	Absorption	Distribution			Metabolism							Excretion	Toxicity
	Intestinal absorption (human)	VDss (human)	BBB permeability	CNS permeability	Substrate		Inhibitor					Total Clearance	AMES toxicity
					CYP								
					2D6	3A4	1A2	2C19	2C9	2D6	3A4		
	Numeric (%Absorbed)	Numeric (Log L/kg)	Numeric (Log BB)	Numeric (Log PS)	Categorical (Yes/No)							Log ml/min/kg	Categorical (Yes/No)
M01	80.66	-0.219	-1.46	-3.17	No	Yes	No	Yes	Yes	No	Yes	0.076	No
M10	88.18	0.319	-1.15	-2.12	No	Yes	No	yes	Yes	No	Yes	0.583	No
M30	91.05	-0.147	-0.16	-2.51	No	No	Yes	Yes	Yes	No	Yes	0.209	No
M38	78.60	-0.270	-1.63	-3.16	No	Yes	No	Yes	Yes	No	Yes	-0.052	No
D01	80.41	-0.129	-1.81	-3.48	No	Yes	No	Yes	Yes	No	Yes	-0.271	No
D02	81.19	-0.012	-1.78	-3.43	No	Yes	No	Yes	Yes	No	Yes	-0.103	No
D03	75.15	0.095	-1.53	-2.92	No	Yes	No	Yes	Yes	No	Yes	0.223	No
D04	77.59	0.174	-1.46	-2.75	No	Yes	No	Yes	Yes	No	Yes	0.371	No
D05	78.46	0.301	-1.30	-2.59	No	Yes	No	Yes	Yes	No	Yes	0.084	No
D07	88.87	0.394	-1.30	-1.82	No	Yes	No	Yes	Yes	No	Yes	-0.360	No
D08	76.04	0.238	-1.37	-2.76	No	Yes	No	Yes	Yes	No	Yes	0.134	No
D09	81.16	0.064	-1.59	-3.28	No	Yes	No	Yes	Yes	No	Yes	-0.094	No
D10	80.32	-0.114	-1.80	-3.45	No	Yes	No	Yes	Yes	No	Yes	-0.281	No
D11	90.97	-0.257	-1.44	-3.19	No	Yes	No	Yes	Yes	No	Yes	-0.317	No
D12	82.19	0.16	-1.20	-3.20	No	Yes	No	Yes	Yes	No	Yes	0.036	No
D14	84.67	0.464	-0.88	-2.41	No	Yes	No	Yes	Yes	No	Yes	-0.227	No
D15	81.88	0.461	-0.90	-2.54	No	Yes	No	Yes	Yes	No	Yes	-0.161	No
D17	73.68	0.237	-1.95	-3.33	No	No	No	Yes	Yes	No	Yes	-0.342	No
D21	85.48	0.204	-1.21	-2.54	No	Yes	No	Yes	Yes	No	Yes	0.226	No
D22	83.57	-0.205	-1.18	-2.63	No	Yes	No	Yes	Yes	No	No	0.280	No

D: Designed compounds, CYP: Cytochrome P

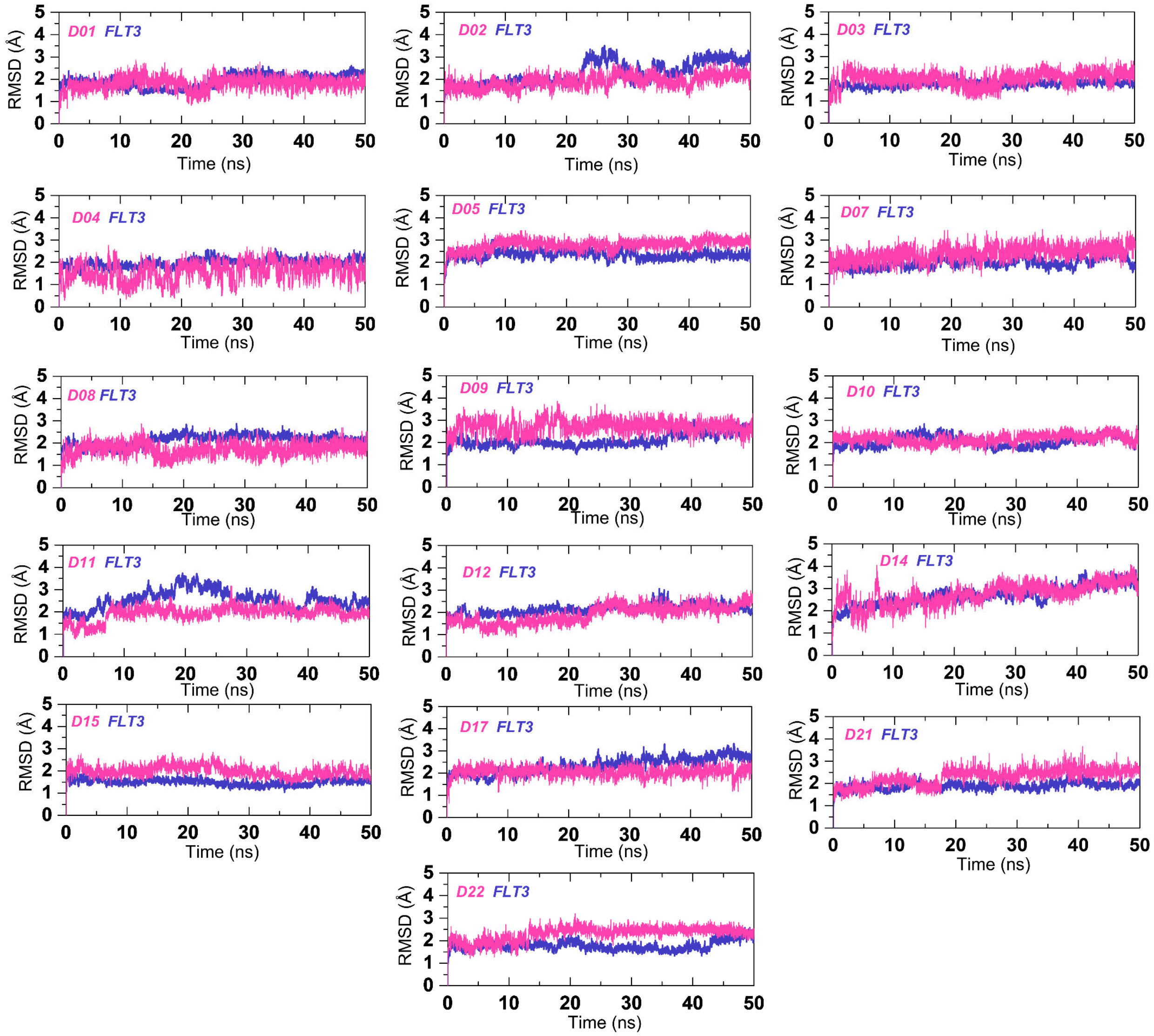

Supplementary Figure S3. RMSD graphs from the MD analysis of the designed compounds.

Supplementary Table S9. Per-residue MM-PB(GB)SA ΔTOTAL binding free energy decomposition in kcal/mol.

Residues	Designed Compounds															
	**D01**	**D02**	**D03**	**D04**	**D05**	**D07**	**D08**	**D09**	**D10**	**D11**	**D12**	**D14**	**D15**	**D17**	**D21**	**D22**
**L576**	-1.05	-1.56	-1.13	-1.32	-	-1.08	-0.98	-1.15	-1.28	-1.48	-1.40	-1.81	-2.52	-1.48	-1.18	-1.70
**L616**	-2.64	-2.38	-2.54	-2.67	-1.74	-2.76	-2.45	-2.53	-1.69	-1.62	-2.33	-2.20	-2.01	-2.34	-2.01	-2.05
**V624**	-1.34	-1.14	-1.37	-1.39	-1.71	-1.35	-	-1.44	-	-	-1.22	-	-0.71	-1.40	-1.59	-1.56
**A642**	-1.20	-1.39	-1.20	-1.21	-1.24	-1.20	-1.05	-1.20	-0.69	-	-1.37	-0.79	-1.10	-1.21	-0.56	-0.63
**K644**	-0.98	-3.27	-1.01	-1.63	-0.73	-1.25	-1.58	1.67	-1.39	-0.52	-2.30	0.60	2.57	-1.96	-0.53	0.16
**E661**	-	-	-	-	-	-	-	-	-	-	-	-	-3.23	-1.42	-2.35	-2.81
**M664**	-0.87	-0.87	-0.38	-0.40	-	-0.80	-	-0.92	-	-	-0.89	-1.89	-0.77	-	-	-
**M665**	-1.49	-2.34	-1.65	-1.32	-1.39	-1.32	-0.82	-2.16	-1.41	-1.93	-2.00	-2.68	-1.13	-1.44	-2.22	-1.29
**L668**	-	-	-	-	-	-	-	-	-	-	1.01	-1.25	-0.40	-	-0.49	-
**I674**	-0.28	-1.03	-0.93	-1.07	-	-1.06	-0.69	-1.11	-0.56	-1.55	-1.33	-1.55	-0.84	-0.67	-1.19	-0.67
**V675**	-3.26	-1.35	-1.66	-1.74	-1.01	-1.64	-3.04	-1.35	-1.96	-2.17	-1.13	-1.09	-1.44	-1.66	-1.46	-1.32
**F691**	-2.67	-2.42	-2.71	-2.66	-2.82	-2.14	-2.77	-2.75	-2.47	-2.67	-2.42	-1.90	-2.29	-2.65	-2.14	-2.04
**Y693**	-1.89	-1.99	-1.82	-1.76	-2.03	-2.07	-1.75	-1.92	-1.11	-1.49	-2.13	-0.51	-	-2.63	-	-
**C694**	-2.97	-2.28	-2.89	-2.77	-1.35	-2.65	-2.37	-2.85	-0.67	-0.57	-2.62	-	-1.21	-2.93	-	-
**G697**	-1.43	-1.78	-1.49	-1.39	-0.92	-1.53	-1.54	-1.50	-1.09	-	-1.80	-0.52	-1.52	-1.76	-	-1.31
**L818**	-1.85	-2.15	-1.93	-2.00	-2.35	-1.95	-2.27	-1.87	-1.91	-1.87	-2.08	-2.19	-2.04	-1.92	-2.03	-1.97
**C828**	-1.20	-1.25	-0.68	-0.77	-2.30	-1.48	-4.03	-0.80	-0.54	-1.13	-1.24	-2.12	-1.72	-0.42	-0.72	-2.03
**D829**	-1.28	-1.42	-2.39	-1.94	-2.50	-1.37	-1.57	-1.03	-1.42	-0.68	-1.11	-0.21	-1.56	-1.12	-1.04	-1.53
**F830**	-1.00	-0.20	-0.88	-1.03	-2.84	0.95	-0.29	-0.76	-2.89	-2.44	-	-0.91	-1.19	-0.75	-1.69	-1.81
**A833**	-	-	-	-	-0.67	-	-	-	-	-	-	-	-	-	-	-
**R834**	-	-	-	-	-	-	-	-	-3.10	-	-	-	-	-	-	-

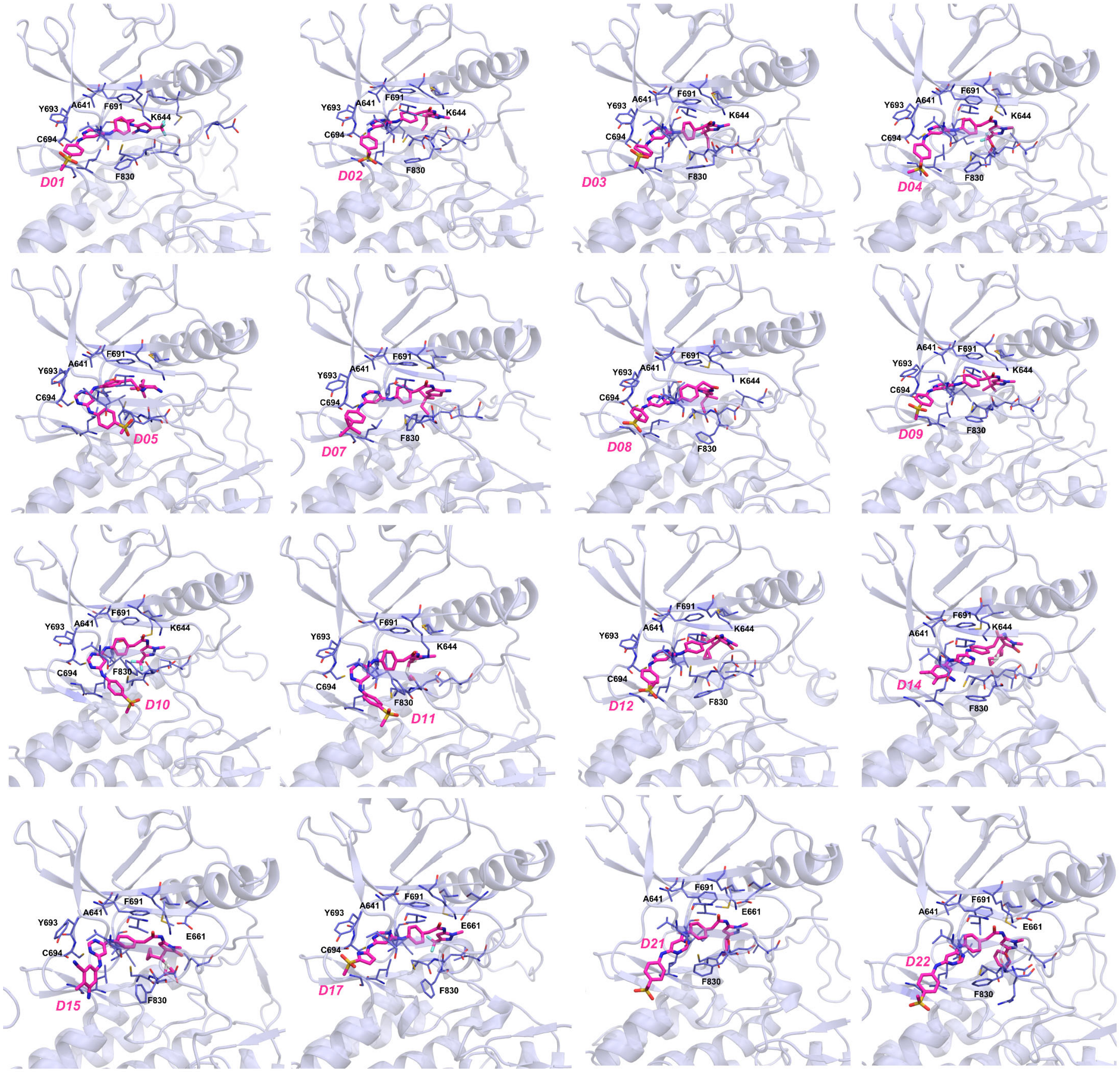

Supplementary Figure S4. Average MD structure of the Designed compounds and FLT3**.**

Methodology:

The external validation of the CoMFA and CoMSIA by test set compounds were crucial step to determine the true predictive power of any QSAR models. The following criteria proposed by Roy et al.[1] , Gramatica et al.[2] and Todeschini et al.[3] were used to externally validate the models. The in-depth analysis process was described in the Supplementary material.[2]

$$0.85 \le k \le 1.15 \text{ or } 0.85 \le k' \le 1.15$$

where, k and k′ are the slopes between the observed vs predicted, and predicted vs observed activity at zero intercept, respectively.

$$|r_0^2 - r'^2_0| < 0.3 \ (1)$$

$$\frac{r^2 - r_0^2}{r^2} < 0.1 \text{ or } \frac{r^2 - r'^2_0}{r^2} < 0.1 \ (2)$$

where, r^2 is the squared correlation coefficient between the predicted and observed

activity values, whereas the r_0^2 and $r_0'^2$ can be expressed as,

$$r_0^2 = 1 - \frac{\Sigma(y_i - k \times \hat{y}_i)^2}{\Sigma(y_i - \bar{y}_i)^2} \ (3)$$

$$r'^2_0 = 1 - \frac{\Sigma(\hat{y}_i - k\prime \times y_i)^2}{\Sigma(\hat{y}_i - \bar{\hat{y}}_i)^2} \ (4).$$

The y_i, $\hat{y}_i$, $\bar{y}_i$, and $\bar{\hat{y}}_i$ are the observed activity, predicted activity, mean value of the observed activity and mean value of the predicted activity respectively.

r_m^2 or $r'^2_m > 0.5$, $\Delta r_m^2 < 0.2$, and $\overline{r_m^2} > 0.5$ can be consider as a good indication of the good predictivity of the QSAR model, where,

$$r_m^2 = r^2 \times (1 - |\sqrt{r^2 - r_0^2}\ |) \ (5)$$

$$r'^2_m = r^2 \times (1 - |\sqrt{r^2 - r'^2_0}\ |) \ (6)$$

$$\Delta r_m^2 = |r_m^2 - r'^2_m| \ (7)$$

$$\overline{r_m^2} = (r_m^2 + r'^2_m) \ (8).$$

Additional parameters, such as Residual Sum Square (RSS) and Mean Absolute Error can be calculated as:

$$MAE = \frac{\sum_{i=1}^{n} |y_i - \hat{y}_i|}{n} \ (9)$$

$$RSS = \sum_{i=1}^{n} (y_i - \hat{y}_i)^2 \ (10)$$

where, y_i and $\hat{y}_i$ is the observed and predicted activity, and n is number of compounds in the test set.

According to Todeschini et al.[3] the predictive correlation coefficient[1-2] matrix (Q_{Fn}^2) can be calculated by following equations:

$$Q_{F1}^2 = 1 - \frac{\sum_{i=1}^{n_{EXT}}(y_i - \hat{y}_i)^2}{\sum_{i=1}^{n_{EXT}}(y_i - \bar{y}_{TR})^2} \quad (11)$$

$$Q_{F2}^2 = 1 - \frac{\sum_{i=1}^{n_{EXT}}(y_i - \hat{y}_i)^2}{\sum_{i=1}^{n_{EXT}}(y_i - \bar{y}_{EXT})^2} \quad (12)$$

$$Q_{F3}^2 = 1 - \frac{\frac{\sum_{i=1}^{n_{EXT}}(y_i - \hat{y}_i)^2}{n_{EXT}}}{\frac{\sum_{i=1}^{n_{EXT}}(y_i - \bar{y}_{TR})^2}{n_{TR}}} \quad (13)$$

However, in LOO methods each training set compound was used once as a test set compound, hence Q_{F1}^2= Q_{F2}^2= Q_{F3}^2, and only Q_{F3}^2 metrics was opted here in this study.

The Concordance Correlation Coefficient (Q_{ccc}^2):

$$Q_{ccc}^2 = \frac{2\sum_{i=1}^{n_{EXT}}((y_i - \overline{y_i})((\hat{y}_i - \bar{\hat{y}}_i)}{\sum_{i=1}^{n_{EXT}}(y_i - \bar{y})^2 + \sum_{i=1}^{n_{EXT}}(\hat{y}_i - \bar{\hat{y}})^2 + n_{EXT}(\bar{y} - \bar{\hat{y}})^2} \quad (14)$$

The applicability domain (AD) analysis of the QSAR models was done using the leverage approach as described in this study[4]. The standardized residuals from the activity values of the training set and test set compounds were plotted against their leverage values in the Williams plot. The detailed methodology was described in the Supplementary materials. The standardized residual (δ) can be calculated as:

$$\delta = \frac{y_i - \hat{y}_i}{\sqrt{\sum_{i=1}^{n}\frac{(y_i - \hat{y}_i)^2}{(n-A-1)}}} \quad (15)$$

The y_i, and $\hat{y}_i$are the the observed activity and predicted activity, n is the number of compounds, A is the number of descriptors.

The leverage h_i is expressed as:

$$h_i = X_i^T(X^TX)^{-1}X_i \quad (i=1,..,n) \ (16)$$

where, X_i stands for the descriptor-row vector of the i-th compound and X_i^T is the transpose of X_i. X is the descriptor matric of the training set compounds, while X^T is the transpose of X.

The warning leverage (h*) was calculated by the following equation:

$$h^* = 2.5\frac{(j+1)}{m} \quad (17)$$

where j is the number of descriptors and m is the number of compounds in the training set. The leverage value of any compound exceeding the warning leverage (h*), as shown with the red dotted line, was denoted as outliers and influenced the model quality.

Reference:

1. Roy, K.; Chakraborty, P.; Mitra, I.; Ojha, P. K.; Kar, S.; Das, R. N., Some case studies on application of "rm2" metrics for judging quality of quantitative structure–activity relationship predictions: emphasis on scaling of response data. *J. Comput. chem.* **2013,** *34* (12), 1071-1082.
2. Gramatica, P.; Sangion, A., A historical excursus on the statistical validation parameters for QSAR models: a clarification concerning metrics and terminology. *J. Chem. Inf. Mod.* **2016,** *56* (6), 1127-1131.
3. Todeschini, R.; Ballabio, D.; Grisoni, F., Beware of unreliable Q 2! A comparative study of regression metrics for predictivity assessment of QSAR models. *J. Chem. Inf. Mod.* **2016,** *56* (10), 1905-1913.
4. Abdizadeh, R.; Hadizadeh, F.; Abdizadeh, T., QSAR analysis of coumarin-based benzamides as histone deacetylase inhibitors using CoMFA, CoMSIA and HQSAR methods. *J. Mol. Struc.* **2020,** *1199*, 126961.
